# Complex Genomic Rearrangements at the *PLP1* Locus Include Triplication and Quadruplication

**DOI:** 10.1371/journal.pgen.1005050

**Published:** 2015-03-06

**Authors:** Christine R. Beck, Claudia M. B. Carvalho, Linda Banser, Tomasz Gambin, Danielle Stubbolo, Bo Yuan, Karen Sperle, Suzanne M. McCahan, Marco Henneke, Pavel Seeman, James Y. Garbern, Grace M. Hobson, James R. Lupski

**Affiliations:** 1 Department of Molecular and Human Genetics, Baylor College of Medicine, Houston, Texas, United States of America; 2 Centro de Pesquisas Rene Rachou- FIOCRUZ, Belo Horizonte, Minas Gerais, Brazil; 3 Nemours Biomedical Research, Alfred I. duPont Hospital for Children, Wilmington, Delaware, United States of America; 4 Jefferson Medical College, Thomas Jefferson University, Philadelphia, Pennsylvania, United States of America; 5 Department of Pediatrics and Adolescent Medicine, Division of Pediatric Neurology, University Medical Center Göttingen, Georg August University, Göttingen, Germany; 6 Department of Pediatric Neurology, DNA Laboratory, 2nd Faculty of Medicine, Charles University and Motol University Hospital, Prague, Czech Republic; 7 University of Rochester Medical Center, Rochester, New York, United States of America; 8 University of Delaware, Department of Biological Sciences, Newark, Delaware, United States of America; 9 Department of Pediatrics and Human Genome Sequencing Center, Baylor College of Medicine, Houston, Texas, United States of America; 10 Texas Children’s Hospital, Houston, Texas, United States of America; University of Pennsylvania, UNITED STATES

## Abstract

Inverted repeats (IRs) can facilitate structural variation as crucibles of genomic rearrangement. Complex duplication—inverted triplication—duplication (DUP-TRP/INV-DUP) rearrangements that contain breakpoint junctions within IRs have been recently associated with both *MECP2* duplication syndrome (MIM#300260) and Pelizaeus-Merzbacher disease (PMD, MIM#312080). We investigated 17 unrelated PMD subjects with copy number gains at the *PLP1* locus including triplication and quadruplication of specific genomic intervals—16/17 were found to have a DUP-TRP/INV-DUP rearrangement product. An IR distal to *PLP1* facilitates DUP-TRP/INV-DUP formation as well as an inversion structural variation found frequently amongst normal individuals. We show that a homology—or homeology—driven replicative mechanism of DNA repair can apparently mediate template switches within stretches of microhomology. Moreover, we provide evidence that quadruplication and potentially higher order amplification of a genomic interval can occur in a manner consistent with rolling circle amplification as predicted by the microhomology-mediated break induced replication (MMBIR) model.

## Author Summary

Genomic architecture, such as direct or inverted repeats, can facilitate structural variation (SV) of the human genome. SV can consist of deletion, duplication, or inversion of a genomic segment, or combinations thereof, the latter referred to as complex genomic rearrangements (CGR). CGR are defined as requiring two or more novel DNA breakpoint junctions. We described a CGR product at the *MECP2* locus with an unusual pattern consisting of an inverted triplicated segment flanked by duplicated segments of the genome. This complex CGR is facilitated by inverted repeats in a process that mechanistically could occur by two template switches mediated by replicative DNA repair. We now investigate the *PLP1* locus and demonstrate that 16/17 CGR independent events present with duplication—inverted triplication—duplication pattern facilitated by two inverted repeats, similar to events involving *MECP2*. We show that the same inverted repeats facilitating CGR formation are also responsible for an inversion polymorphism observed frequently in the normal population. Intriguingly, one CGR was found to have a quadruplication resulting in the presence of four copies of a genomic segment. Breakpoint studies suggest this quadruplication occurred in a manner consistent with rolling circle amplification as predicted by previously postulated models.

## Introduction

Inverted repeats (IRs) are a common architectural feature within the human genome and can predispose loci to rearrangement [1–3]. An IR-mediated inversion that disrupts the *Factor VIII* gene causes ~45% of severe hemophilia A cases [4]. The importance of IRs to human genomic rearrangements and resultant genomic disorders and the expanded scope by which IRs can facilitate genomic change are now apparent [2,3,5–7]. The abundance of inverted low copy repeats (LCRs) or segmental duplications genome-wide suggests that ~12% of the genome may be susceptible to inversion mediated by IRs [2]. Fosmid paired-end sequencing of 8 human genomes from diverse populations shows that ~50–100 large genomic inversions not represented in the human genome reference sequence are present in the personal genome of each individual. In total, 224 non-redundant inversions were identified in 8 genomes; these events are primarily mediated by larger blocks of homology [8]. Earlier work provided experimental evidence for genome-wide inversions and suggested these can occur somatically and with aging [9]. Moreover, inverted repetitive regions that are smaller than conventional LCRs, designated self-chains, are also associated with genomic instability furthering the impact of IRs on both structural human differences and phenotypes [3].

Recently, IRs were shown to mediate complex duplication—inverted triplication—duplication (DUP-TRP/INV-DUP) rearrangements, leading to *MECP2* duplication syndrome (MIM#300260), Duchenne Muscular Dystrophy (MIM#310200), *VIPR2* triplication, *CHRNA7* triplication, and Pelizaeus-Merzbacher disease (PMD, MIM#312080) [1,10–13]. The mechanisms for such complex genomic rearrangements (CGRs) have only begun to be elucidated.

Genomic rearrangements leading to the duplication of the X-linked *proteolipid protein 1* (*PLP1*) gene are the major mutational cause for PMD and explain ~80% of patients; point mutations in *PLP1* occur less frequently, and higher copy number gains (*e*.*g*. triplications) and deletions are rare [14–17]. CGR can cause PMD by duplicating *PLP1* via a mechanism that results in a DUP-TRP/INV-DUP structure [1]. Consistent with a gene dosage hypothesis, and as established for both homozygous duplication [18] and heterozygous triplication [19] at the CMT1A locus, triplication of *PLP1* can lead to a more severe form of PMD than duplication [13,14].

Using high-density array comparative genomic hybridization (aCGH), DUP-TRP/INV-DUP rearrangements primarily contain one variable breakpoint at the proximal (centromeric) end [1]; however, distal breakpoints for the triplication to duplication and duplication to normal copy number transitions cluster at inverted LCRs distal to *MECP2* [1]. The proposed mechanism for these CGR involved a two-step process: i) break-induced replication (BIR) within homologous regions of the inverted LCRs forming a breakpoint junction (Jct1) and ii) microhomology-mediated BIR (MMBIR) or non-homologous end-joining forming a second junction (Jct2). Mutational signatures observed at the latter junction include microhomology, templated insertions, and increased point mutation frequency [1,20]. However, in both *MECP2* and *PLP1* DUP-TRP/INV-DUP rearrangements, delineation of unique breakpoint junctions within the IR has been hampered by the complexity of large blocks of homologous sequences creating challenges to mapping Jct1 at base pair resolution.

To further investigate mechanisms for CGR formation we analyzed a cohort of 17 unrelated PMD patients with copy number gains at the *PLP1* locus, including duplications, triplications and quadruplication. Analysis of phenotypically normal individuals elucidated a common inversion polymorphism associated with the IRs distal to *PLP1*. Southern blotting experiments established an estimated frequency for the inversion. We postulated and confirmed that the LCR substrates responsible for the inversion are also responsible for one breakpoint junction (Jct 1) in each PMD associated CGR. Additionally, we document a DUP-TRP/INV-DUP rearrangement product structure at the *PLP1* locus in the personal genomes of 16 subjects with PMD and provide evidence that such CGR can occur by replicative mechanisms [21]. Finally, we investigated the quadruplication of a genomic segment proximal to *PLP1* and found the potential mechanism of formation to be consistent with rolling-circle replication leading to amplification—a mechanism predicted by the MMBIR model [22].

## Results

### Inversion Polymorphism Discovery, Frequency and Recurrence

**Fig 1:**
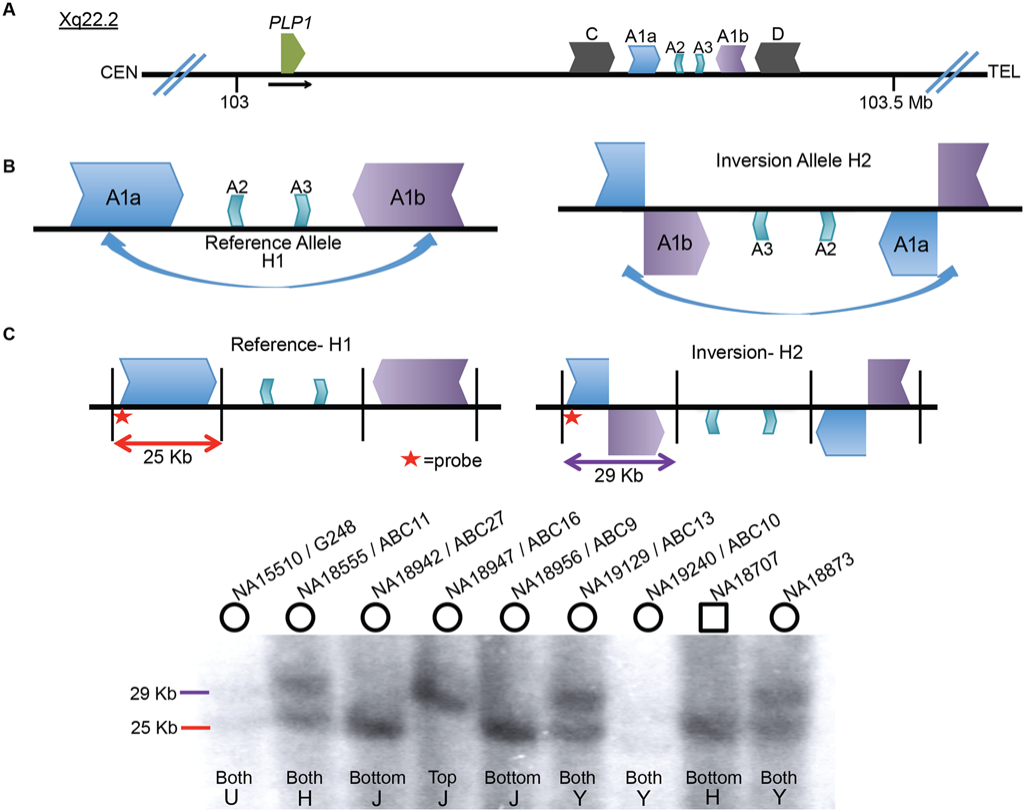
Inversion discovery and frequency between A1a and A1b. **A**) A depiction of the *PLP1* genomic region on chromosome Xq22.2. *PLP1* is proximal to the LCR structures and the black horizontal arrow indicates direction of transcription. There are three IRs present at chrX:103,171,387–103,359,682: the outer C and D repeats, middle A1a and A1b repeats, and inner A2 and A3 repeats [23,24]. The gene and the IR structures are separated by ~150 kb. **B**) A common inversion, discovered in the HGSV resource, is depicted between the A1a and A1b repeats. This inversion was present in at least 5/9 of individuals in the fosmid resource, and is possible in three additional people (S1 Fig). **C**) A Southern blotting scheme to distinguish between reference and inversion alleles is depicted, with A1a, A1b, A2 and A3 represented as above. Female genomes have two alleles, and phenotypically normal males have one allele (blot quantitated in S1 Table). Digestion with BssSI (depicted by black vertical lines) and detection with a probe proximal to LCR A1a (indicated by red star) should distinguish reference (25 kb, red) from inverted (29 kb, purple) alleles. Nine individuals from the HapMap population were studied for inversion via Southern blotting. Gender of the individual is indicated by circles (female) or squares (male) above the blot. DNA identifiers (NA numbers) are consistent with Coriell names (http://ccr.coriell.org/), and fosmid libraries (ABC library identifiers) are as in Kidd et al. [49]. The population of origin for each individual and the genotypes are indicated at the bottom of the blot. H = Han Chinese, J = Japanese, Y = Yoruban, and U = unknown. Genotyping for 8 additional individuals is depicted in S3 Fig.

The 186 kb genomic interval (ChrX: 103,172,000–103,358,000 in hg19) located ~150 kb distal to *PLP1* contains a complex genomic architecture in the haploid reference genome. This region consists of an array of IRs, with the ~40 kb outer C and D repeats having ~93% identity, the middle A1a and A1b repeats ~20 kb in size and ~99% identical, and the innermost ~10 kb A2 and A3 repeats showing ~87% identity both with each other and with A1a and A1b (Fig. 1A) [16,23,24]. The IR architecture predicts the potential for inversion mediated by non-allelic homologous recombination (NAHR), resulting in at least two structural haplotypes, analogous to the H1 and H2 structural variant (SV) alleles at the *MECP2* locus [1]. Indeed, *in silico* analysis of the human genome SV track from the UCSC Genome Browser (www.genome.ucsc.edu) suggests the existence of such an SV allele [25]. The browser track indicates fosmids consistent with inversions spanning both of the A1a and A1b LCRs in 5 of 9 individuals (S1 Fig) [8,26]. These data indicate that there was an inversion between A1a and A1b LCRs and that the inversion haplotype exists at a relatively high allele frequency as a non-pathogenic rearrangement in HapMap individuals (Fig. 1B). Further investigation mapped the apparent ectopic crossover for the NAHR-mediated inversion in a fosmid from the G248 library to nucleotide-level resolution (S2 Fig).

To directly examine the inversion SV polymorphism between A1a and A1b, we designed a Southern blotting assay and genotyped multiple individuals from different populations of origin for reference (arbitrarily designated H1) or inversion (H2) structural haplotypes. The scheme of the assay is depicted in Fig. 1C, wherein Southern analysis leads to predicted visible fragments of 25 kb for H1 and/or 29 kb for H2. As the rearrangement is on the X chromosome, males should have only one allele, and females, two. Genotyping 17 individuals (including 3 males) with this assay discerned 31 haplotypes of the X chromosome (Figs. 1C, S3, and S1 Table). The frequencies of structural haplotypes were 13/31 H2 (~42%) and 18/31 H1 (~58%), with 4 individuals hemizygous or homozygous for H2 and 7 for H1. The remaining 6 females were heterozygous for both H1 and H2. The 17 individuals were of Japanese, CEPH Northern European, Han Chinese, Yoruban and unknown populations of origin, and all populations contained both H1 and H2 structural haplotypes (S2 Table).

We hypothesized that the similarity between LCRs A1a and A1b and their relatively large length and proximity (~20 kb repeats of ~99% identity separated by ~50 kb) could predispose to recurrent events [27,28]. We analyzed the genomic region encompassing the two LCRs and identified multiple adjacent single nucleotide polymorphisms (SNPs) spanning the region in linkage disequilibrium and delineating a haplotype block extending for ~0.5 Mb with a recombination rate of 0.3 centimorgans per Mb. The two SNP haplotype blocks were evenly distributed between the 14 different populations from the 1,000 genomes project [29]. Superimposing Southern blotting results for individuals homozygous or hemizygous for SV haplotypes on top of the SNP haplotypes enabled phasing; 6/7 inversion (H2) alleles were on one SNP haplotype and 1/7 was on the other (belonging to individual NA18947, S4 Fig), whereas homozygous H1 alleles occurred on either SNP haplotype. Heterozygous calls are uninformative, as the structural haplotype information cannot be phased to the SNP data. These data suggest that the inversion is likely recurrent in the population and makes population estimation of the structural variant using SNP genotyping unlikely to reflect the true population frequency.

### Breakpoint Mapping of *PLP1* Rearrangements

**Fig 2:**
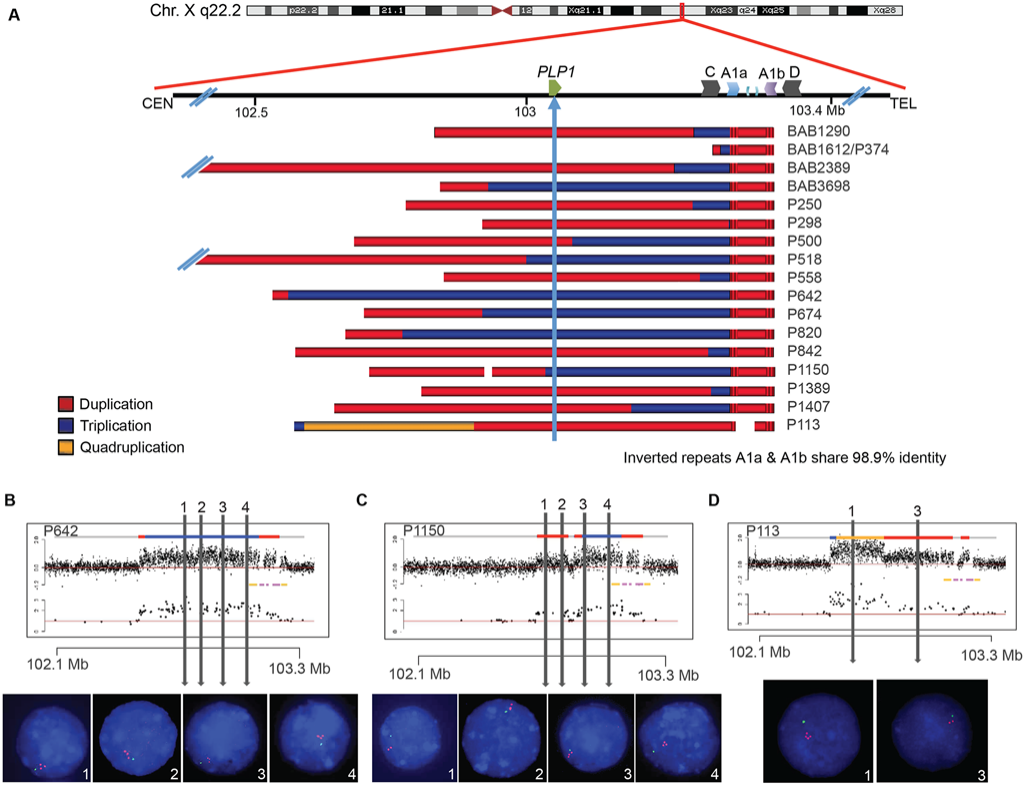
Complex rearrangements involving *PLP1* have clustered breakpoints in IRs. **A**) 17 individuals are depicted with schematics of their array results. Duplications are indicated in red, triplications are indicated in blue, and the quadruplication is indicated in gold. Coordinates and LCR blocks are indicated in the cartoon above the array results (A2 and A3 are unlabeled). P1150 and P113 contain copy number neutral segments within the rearrangement, leading to a copy number of 1 indicated by white space in the array schematic (see also S5 and S6 Figs). **B**) FISH results for P642, depicting a large triplication of the *PLP1* region, including the gene. All three copies of the four probes are on Xq22.2. Array data are at the top and qPCR data are below, with LCRs indicated in yellow and pink. Colors for copy number in array data are as in panel A. Probe locations for FISH denoted by vertical lines. **C**) FISH results for P1150, similar to those in B. The figure depicts duplication of the two proximal probes 1 and 2 and triplication of the distal 3 and 4 probes, as proposed by the array data above. **D**) FISH results for patient P113, depicting a quadruplication/duplication rearrangement of the region. High-resolution aCGH showed greater complexities, including a small triplication and a normal copy number segment within the duplication (See Figs. 5 and S5). In all FISH studies, nuclei are stained with DAPI, and a marker for the short arm of chromosome X, AL353698, is shown in green. Vertical arrows with numbers in the array data above indicate positions of FISH probes throughout the *PLP1* region.

Sixteen patients with PMD and one diagnosed with spastic paraplegia type two (SPG2; MIM#312920) were examined by aCGH for copy number variation (CNV) in *PLP1* and the surrounding genomic region. A schematic of CNV observed in the personal genomes from 17 patients is depicted in Fig. 2A. *PLP1* duplications were detected in 10 patients (BAB1290, BAB2389, P250, P298, P500, P558, P842, P1389, P1407 and P113), whereas triplications were detected in 6 patients (BAB3698, P518, P642, P674, P820, and P1150). The one SPG2 patient, BAB1612/P374 has been described previously, and the phenotype of this individual is ascribed to a potential position effect [30]. The distal breakpoints in all subjects appear to cluster in approximately the same genomic location; however, there are few probes on the arrays that can specify unique loci within the C/D, A1a/A1b, and A2/A3 LCRs due to the repeat nature of the region. Thus, determining the precise LCRs involved in the breakpoints required alternate mapping approaches.

Array and semi-quantitative PCR data, summarized in Fig. 2A (see also S5 Fig and Table 1), indicate that the region of rearrangement spans from 145 kb (BAB1612/P374) to ~4,000 kb (BAB2389). Triplicated genomic segments range in size from 254 bp (P298/P255) to 575 kb (P642). Proximal triplication and duplication copy number transitions differed in each individual and were not located within LCRs. The distal copy number transitions group within a 100 kb region of uncertainty as described above. The triplication present in P298/P255 was too small to be detected by aCGH; however, a 254 bp triplicated genomic segment was detected both by amplification and sequence analysis with unique flanking primers and by quantitative PCR (S6 Fig).

**Table 1:** Unique Junctions (Jct2) in DUP-TRP/INV-DUP rearrangements.

Patient		Array	Triplication start point	Triplication size (bp)	Proximal dup. start point	Duplication size (bp)	Microhomology^a^	Inserted Sequence ()^b^	Blunt end junctions	Chimeric Element(s)	Junction in repetitive element or LCR^c^
1	P250	NimbleGen	103145195	78476	102866025	458310	**-gagTTGActg-**	**-g(a)gTTGActg-**	N/A	N/A	MER101 **//** N/A
2	P298	Affymetrix	103223417	254	102943388	380947	**-aacAAtgc-**	N/A	N/A	N/A	N/A
3	BAB 1612/P374	Affymetrix	103198235	25436	103179140	145195	**-aagAGGTtcc-**	N/A	N/A	N/A	L2c (LCR) **//** L2c (same LCR)
4	P500	NimbleGen/Agilent	103073314	150357	102723607	600728	**-ctcTAGctt… …aggtcatttat-**	**93bp of 102739366(+)…ggt(c)attt(a)t-**	N/A	N/A	L1ME4a **//** HAL1b **//** L2
5	P518	NimbleGen	103004306	219365	102324702	999633	**-ggcCT…ACcat… …atTA…AAtta-**	**488bp of 103003940(+)**	N/A	*Alu*S (34bp) / *Alu*S (14bp)	*Alu*Sq **//** *Alu*Sx…*Alu*Sq **//** *Alu*Sg
6	P558	NimbleGen	103149848	73823	102804340	519995	N/A	N/A	**-ttgg/tctg-**	N/A	L1PA2 **//** MIR
7	P642	NimbleGen	102648490	575181	102627018	697317	**-ggtAGcct-**	N/A	N/A	N/A	L1M4c **//** N/A
8	P674	NimbleGen	102963070	260601	102793421	530914	**-actAggc-**	N/A	N/A	N/A	N/A
9	P820	NimbleGen	102847223	376448	102770530	553805	**-tagGAGaAAggt-**	N/A	N/A	N/A	L1PA7 **//** MIRb
10	P842	NimbleGen	103196894	26777	102642482	681853	N/A	N/A	**-gaaa/ttct-**	N/A	L1ME3B **//** *Alu*Sc
11	P1150	NimbleGen	103019045	204626	102803592	520743	**-ttcAGgaa-**	N/A	N/A	N/A	HERVH **//** L1MA9
12	P1389	Affymetrix	103180817	42854	102885051	439284	**-ggaGcag-**	N/A	N/A	N/A	LCR **//** N/A
13	P1407	Affymetrix/Agilent	103088384	135287	102744808	579527	**-agcaTGctca-**	N/A	N/A	N/A	L2a **//** N/A
14	BAB1290	Agilent	103128008	95663	102528713	795622	**-atgATTTagg… …ttttatagc-**	**43bp of 102550108(+), …ttt(tat)agc-**	N/A	N/A	L1PA3 **//** L1MB4 **//** L1M5
15	BAB2389	Agilent	103081145	142528	99388802	3935533	**-aaaCtgg… …cttTATact-**	**57bp of 103093622(-)**	N/A	N/A	N/A **//** N/A **//** L1MEc
16	BAB3698	Agilent	102995680	227991	102944787	379548	**-taggGATGG… …GACCTcagg-**	N/A	N/A	*Alu*Sx (47 bp identity)	*Alu*Sx **//** *Alu*Sx

All DUP-TRP/INV-DUP rearrangements at Xq22.2 have an approximate triplication endpoint of ChrX:103223671 and distal duplication endpoint of ChrX:103324335, and all coordinates are with respect to hg19/GRCh37. The breakpoint junctions are detailed in S6 Fig. N/A stands for not applicable (there is no microhomology, inserted sequence, blunt junction, chimeric element, nor repetitive element with this breakpoint or at the specified side of the junction)

^a^Microhomology at breakpoint is indicated with capital letters. Dashes indicate sequences surrounding the junctions, and … indicates either inserted sequences leading to two junctions or extended homology due to a chimeric element.

^b^Inserted sequence/additional template switches at the breakpoint junctions. Small insertions are indicated in brackets (), whereas larger insertions are indicated with the coordinates of the inserted sequences and the strand (+ or-).

^c^Repetitive elements or low copy repeats (LCRs) present at the breakpoint junctions are indicated with the repeat name. // indicates the junction. Only P518 and BAB3698 contain junctions resulting in chimeric elements.

FISH was performed on nuclei prepared from peripheral blood lymphocytes from P642, P1150, and P113. This independently corroborated interpretation of array and semi-quantitative PCR data using an orthogonal experimental approach. Moreover, FISH determined whether extra copies of the genomic segments were located in or near the *PLP1* locus as opposed to elsewhere in the genome; arrays determine neither the orientation nor the position of a copy number segment, but only specify the genomic segment that underwent a gain in copy number (Fig. 2B-D). Interphase nuclei of patients P642 and P1150 showed, as expected, one green control probe signal and revealed three closely-spaced red *PLP1* probe signals indicating triplication at the *PLP1* locus; P113 had two red *PLP1* signals indicating duplication at that locus, but four proximal probe signals confirmed the additional quadruplication (Fig. 2D). Metaphase spreads of all 3 patients gave one green control probe signal, one red presumably merged *PLP1* probe signal on the X chromosome, and no signals on other chromosomes, also indicating that the triplications and duplications were at the *PLP1* locus rather than being located elsewhere in the genome and that the triplications were too small to resolve on metaphase chromosomes.

### Marker Genotypes Suggest Intra-Chromosomal Events

We investigated haplotypes using genetic markers, 2 short tandem repeats (STRs) and 9 SNPs, mapping over a 258 kb region of the duplications with 4 markers mapping within *PLP1* and the remainder distal to it. We observed that 12 of 13 patients tested (P250, P255/298, BAB1612/P374, P500, P518, P558, P642, P674, P820, P842, P1150, P1389, and P1407), were monomorphic displaying only one form for each marker genotype (S3 Table). The DNA from BAB1612/P374 was only interrogated at the 7 sites distal to *PLP1*, since this is where his triplicated/duplicated region lies. In this subject, only one form was detected for all markers except the STR furthest distal to *PLP1* where two were detected. The finding of an absence of bi-allelic loci in these multi-copy regions of X is most parsimoniously explained by the occurrence of intra-chromosomal rearrangement events, as has also been observed for DUP-TRP/INV-DUP rearrangements at the *MECP2* locus [20].

### Junction Analyses of Proximal Breakpoints

In P1150, we obtained a breakpoint junction between the proximal (centromeric) endpoint of the rearrangement and the proximal end of the triplicated region via inverse PCR. We then hypothesized that our other patients with duplication-triplication-duplication copy number changes could potentially have the same CGR product structure and explored this hypothesis by long-range PCR on each individual personal genome. We were able to amplify and sequence across the proximal breakpoint junction in all 16 patients (Table 1). The 16 junctions each indicate that the triplicated region is inverted with respect to the proximal duplication region (S6 Fig); in 6 cases the triplication encompasses *PLP1*. This is a potentially analogous rearrangement structure to that previously described for *MECP2* CGRs; therefore, we denote the non-recurrent junctions in Table 1 as Jct2 [1].

We had previously mapped and sequenced across the duplication breakpoint junction of patient P255, who had a 254 bp inverted duplication [24]. We no longer had DNA from P255 to interrogate the copy number of the region; therefore, we tested an affected family member, P298, by qPCR and found, as anticipated, that the region is triplicated.

The Jct2 sequences in the 16 patients are shown in Table 1. Fourteen patients contain one or more breakpoint junctions displaying microhomology. Patients P558 and P842 have blunt junctions. In 13 of the patients, endpoints at Jct2 are in repetitive element sequences, and in P1389, one end was in an LCR (Table 1). Patient BAB1612/P374 contained a LINE2-mediated event (L2/L2, both within the same LCR) that did not result in a chimeric element. Patients P518 and BAB3698 contain chimeric *AluS* elements formed in the generation of this junction. In BAB3698, there are 47 bp of identity between the two *AluSx* elements at the transition from triplication to duplication. In P518, the rearrangement occurs through the formation of two *AluS* chimeric junctions, the first (from proximal to middle segment) in the same orientation in 14 bp of identity, and the second (from middle segment to triplication) in 34 bp of identity (see S6 Fig). This complex breakpoint junction contains a segment of 488 bp that consists of an *AluSx3*, an L1 sequence (L1ME), and an *AluSq2*. Interestingly, the distal (triplication) to middle junction occurs between these two *Alu* elements that are only separated by 310 bp, suggesting a potential U-turn caused by inverted *Alus* within close proximity [31,32], similar to the situation in Jct1 but mediated by short *Alu* sequences instead of LCRs (S6 Fig). The breakpoint junction mutational signatures are consistent with replicative mechanisms such as MMBIR or a homeologous (near homologous) recombination event between similar *Alu* elements at each instance of Jct2 [22,33,34].

Complexities that included an additional template switch were observed in Jct2 from individuals P500, P518, BAB1290 and BAB2389 (Table 1 and S6 Fig). Such events have been postulated to reflect reduced processivity of the replisome mediating MMBIR during initial template switching [22]. We also amplified across a breakpoint junction present in P1150, indicating a 27 kb deletion on one of the duplicated copies (Figs. 2 and S5). At that junction, there is a bp of microhomology (S6 Fig). The overall findings for Jct2 are consistent with both long distance template switching and a microhomology-mediated mechanistic process such as FoSTeS/MMBIR [21,22].

### DUP-TRP/INV-DUP Distal Breakpoints

After Jct2 was determined for the 16 patients, we hypothesized that a likely genomic arrangement consistent with this junction was one in which one copy of the triplicated region was situated in an inverse orientation between the two copies of the duplicated region and that the other two copies of the triplication were embedded within the duplicated regions, *i*.*e*. a DUP-TRP/INV-DUP structure [1].

**Fig 3:**
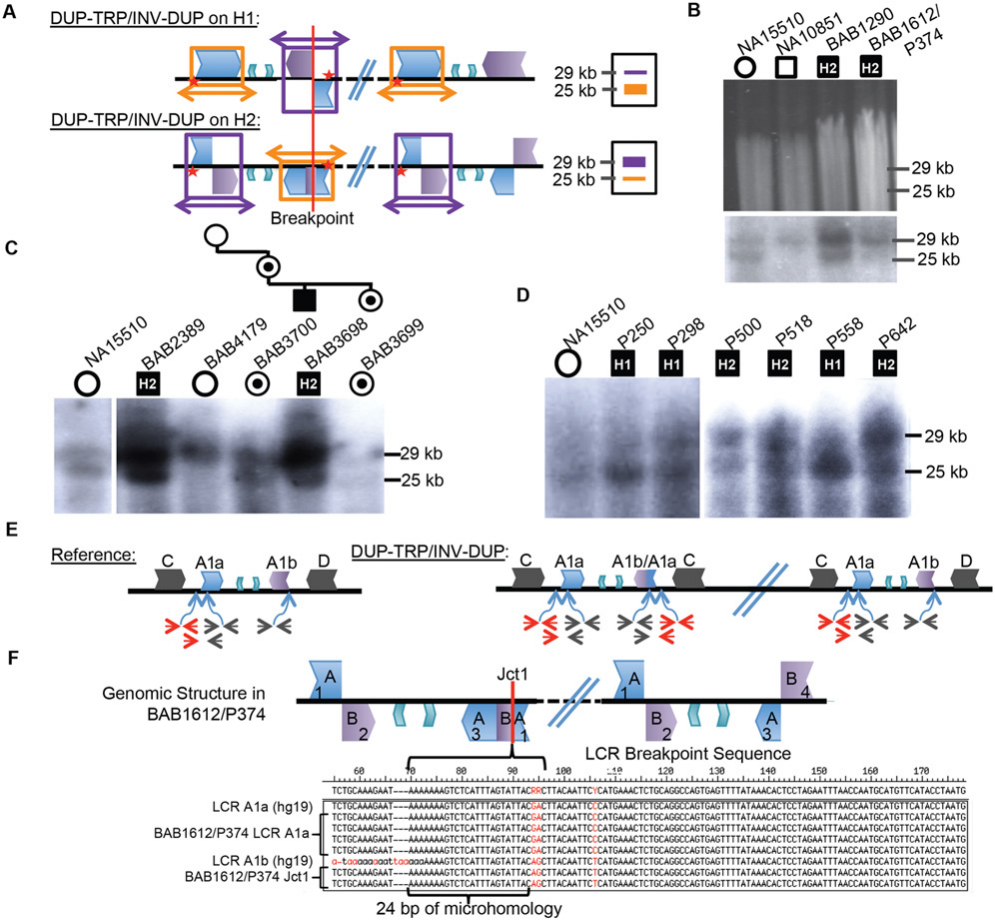
CGRs at the *PLP1* locus use A1a and A1b repeats. **A**) Southern scheme from Fig. 1 applied to a DUP-TRP/INV-DUP rearrangement results in either two copies of a 25 kb band and one of a 29 kb band if the rearrangement occurred on H1 (shown at the top), or the reciprocal copy dosage if the rearrangement occurred on H2 (bottom). Colors for LCRs are as in Fig. 1. **B**) Digested DNA from 2 control individuals (NA15510 and NA10851) and two PMD/SPG2 patients (BAB1290 and BAB1612/P374, respectively). Southern below depicts control individuals have expected, gender appropriate 2 and 1 copies, and affected males have three copies, with dosage of 2:1 H2:H1, indicating the rearrangement likely occurred on H2 (indicated within the black square for each patient- data for this blot and panels C and D are quantitated in S4 Table). **C**) BAB2389 and BAB3698 may also have rearrangements on the inverted allele (H2, indicated at the top of the image). BAB3698 is depicted with his carrier mother, sister (BAB3700 and BAB3699, indicated by a dot in a circle), and non-carrier grandmother (BAB4179). The grandfather was unaffected and unavailable for study. **D**) P250, P298, and P558 all likely contain rearrangements on H1 (~1:2 ratio of H2:H1) and P500, P518, and P642 contain rearrangements on H2. All rearrangement progenitor haplotypes are indicated for the patients above the Southern blot. **E**) The reference genomic structure of H1 is shown (inner A2 and A3 repeats are unlabeled). The qPCR primer pairs amplify a unique region outside of the A1a LCR (in red), inside of both A1a and A1b LCRs (in black) or from the A1a LCR to a unique region outside (red/black pair below). These will give rise to one copy (red pair and red/black pair) or two copies (black pair) in a non-rearranged X chromosome in a male individual. DUP-TRP/INV-DUP (on right) in an H1 haplotype will give rise to four copies amplified by the black pair (2x normal control) and three copies by the red pair and red/black pair (3X normal control) (S7 Fig). **F**) Analysis of Jct1 has successfully cloned one breakpoint in BAB1612/P374. The structure of the LCR-mediated rearrangement on H2 is depicted at the top (A1a and A1b are simplified to “A” and “B” and inner A2 and A3 repeats are unlabeled). Overlapping clones for each region of the two LCRs were generated (numbered 1–4, S8 Fig), and results for BAB1612/P374 were obtained for section 1 clones that both contain and lack the breakpoint. Multiple clones from this region are depicted along with the reference sequences for LCRs A1a and A1b below. The breakpoint from individual BAB1612/P374 occurred in stretch of 24bp of microhomology (bracket-denoted region).

Patients with presumed DUP-TRP/INV-DUP rearrangements with sufficient DNA available were subjected to Southern blotting (10/16 total) to examine whether the same repeats involved in the common inversion polymorphism are also involved in the CGR and to investigate on which structural haplotype the rearrangement occurred. The Southern scheme in Fig. 1C was used to analyze patient DNAs; however, in a male with PMD caused by DUP-TRP/INV-DUP involving the A1a and A1b repeats, the Southern blot does not reflect the normal copy number of one allele of the X chromosome (either H1 or H2) (Fig. 3A, S4 Table). Instead, the rearrangement gives rise to two copies of the original haplotype plus an additional “flipped” haplotype in an affected individual with DUP-TRP/INV-DUP leading to PMD, similar to the observation described for the *MECP2* locus [1]. This assay can presumably distinguish the SV haplotype on which the genomic rearrangement occurred. A representative gel and labeled blot are shown in Fig. 3B, with the dosage of the bands indicating that subjects BAB1290 and BAB1612/P374 both carried the inversion H2 structural haplotype prior to the rearrangement.

Interestingly, the 10 individuals examined by this assay appeared to use the A1a and A1b LCRs as the substrates for their rearrangements, in spite of two other IRs being located in close proximity (BIR between C/D would lead to duplication of H1 or H2 and A2/A3 would lead to triplication) (Figs. 1A, 3C and D). Individuals BAB1290, BAB1612/P374, BAB2389, BAB3698, P500, P518, and P642 all contained a rearrangement that had occurred on the inverted H2 allele, while P250, P298, and P558 had a Southern blot result indicating the rearrangement occurred on an H1 haplotype (S4 Table).

A three-generation family was studied in which the two maternal grandparents were unaffected, and subsequent Southern blotting and aCGH data indicated that the grandmother (BAB4179) was not a carrier and that she had two copies of the inverted H2 locus (Figs. 3C and S5). The grandfather was unavailable, but did not have PMD; therefore, the *de novo* rearrangement can be inferred to have occurred in between the grandparent and the maternal generation. The mother (BAB3700) was a carrier of the rearrangement and had equal dosage of H1 and H2 on a Southern Blot. The affected son (BAB3698) had Southern results consistent with rearrangement on H2, and his carrier sister (BAB3699) had similar results to the mother. These findings are consistent with the *de novo* DUP-TRP/INV-DUP occurring in association with “flipping” the H2 haplotype to an H1 haplotype, a mechanism similar to that observed for CGRs at the *MECP2* locus (Fig. 3A) [1]. The assay results in this family are most parsimonious with the rearrangement occurring on one of the grandmother’s inversion-containing alleles (H2), and having balanced copy number in BAB3700 and BAB3699 due to the additional allele being a reference (H1) 25 kb band. This would result in a 2:2 dosage of 29 kb:25 kb bands on the Southern blot, which we observe in both BAB3700 and 3699 (Fig. 3C and S4 Table).

### Breakpoint Junctions or Crossovers Within LCRs

As Jct1 occurs within the LCR region distal to *PLP1*, the junctional products are not readily amplified and sequenced by long PCR with primers anchored to unique flanking sequence. We adopted an alternative strategy to complement the Southern blotting assay above. Using a semi-quantitative PCR approach, we first confirmed that each of the patients has duplication of A1a and A1b LCRs (black primer pair in Fig. 3E) and triplication of a region proximal to A1a (red primer pair and black/red primer pair in Figs. 3E, S7). This PCR approach independently verified the Southern Blot results and suggested a crossover breakpoint within the A1a or A1a/A1b chimera present on H2 (Fig. 3E). We attempted to more narrowly define the crossover region in our patients by using sequence differences between the LCRs (paralogous sequence variants or PSVs), but patients appeared to lack apparent PSVs between A1a and A1b that were at the corresponding genomic locations in the hg19 reference sequence [35].

To determine sequences across Jct1, we designed a PCR-cloning assay that allowed us to amplify large (~12–16 kb), overlapping portions of both A1a and A1b LCRs that are implicated in the rearrangements [35] (Figs. 3F, S8). Three individuals were subjected to this analysis (BAB1612/P374, BAB2389, and BAB1290), however BAB2389 and BAB1290 appear to have Jct1 within a large region of identity (>8 kb) in the center of the LCR that lacks PSVs between cloned segments; therefore, further refinement of the breakpoint junction was intractable using this method. Additionally, in P255/298, a PCR approach using one primer at the proximal duplication junction and one within the LCR corroborated that the breakpoint indeed occurred within this >8kb stretch of identity.

In contrast to the three other individuals for whom we sought to find Jct1 at base pair resolution, in BAB1612/P374 we were able to detect an LCR-mediated breakpoint within 24 bp of microhomology flanked by A1a and A1b sequences (Fig. 3F). The point of crossover within this sequence was confirmed by direct PCR amplification and sequence analysis from genomic DNA followed by comparison to the PSVs present on cloned A1a and A1b sequences from the same individual; its identification elucidates Jct1 within an LCR, a heretofore un-investigated junction at the nucleotide level of resolution.

### DUP-TRP/INV-DUP Rearrangement Structure

**Fig 4:**
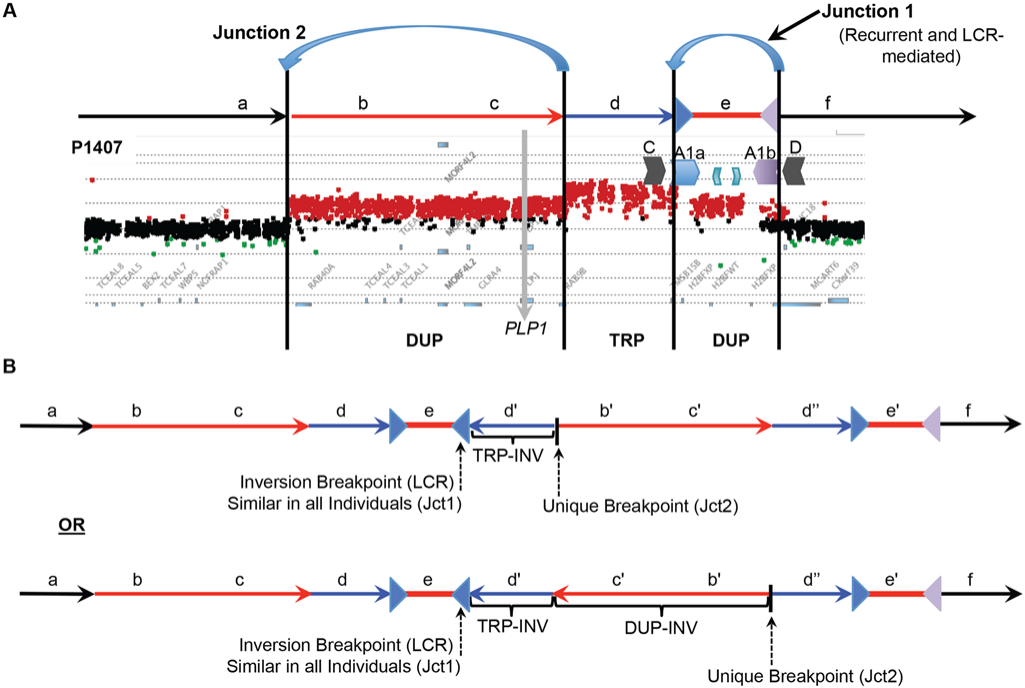
DUP-TRP/INV-DUP structures at the *PLP1* locus. **A**) The aCGH results from patient P1407 showing a duplication of *PLP1* and a distal triplication-duplication structure are shown at the top, with duplications in red and triplication in blue. The relative genomic regions are labeled with letters to distinguish their relative positions within the CGR. The IRs flanking segment e (A1a and A1b) are denoted by inverted blue and purple triangles, respectively. **B**) Two potential structures of the generalized DUP-TRP/INV-DUP rearrangement that are consistent with Jct2 sequencing are shown in the lower panel of the figure in relation to the canonical genomic structure at the top. Here, the unique proximal breakpoint junction location that differs between patients, the LCR-mediated distal inversion breakpoint junction, and the inverted triplication region are seen. Letters followed by a prime symbol indicate duplicated segments. Two prime symbols indicate the triplicated segment.

The DUP-TRP/INV-DUP structure hypothesized for these 16 individuals postulates that although there are 4 copy number transitions in these patients, there are only two breakpoint junctions (Fig. 4A). We have sequenced Jct2 in all 16 patients; Southern blotting and quantitative PCR were used to determine Jct1, and direct junction sequencing was successful for BAB1612/P374 (Figs. 3, 4 and S6). Additionally, due to the small size (~ 254 bp) of the triplication in P255/298, a PCR approach using one primer at the proximal duplication junction and one within the LCR validated the overall structure of this rearrangement as DUP-TRP/INV-DUP.

### Quadruplication by Rolling-Circle Amplification

**Fig 5:**
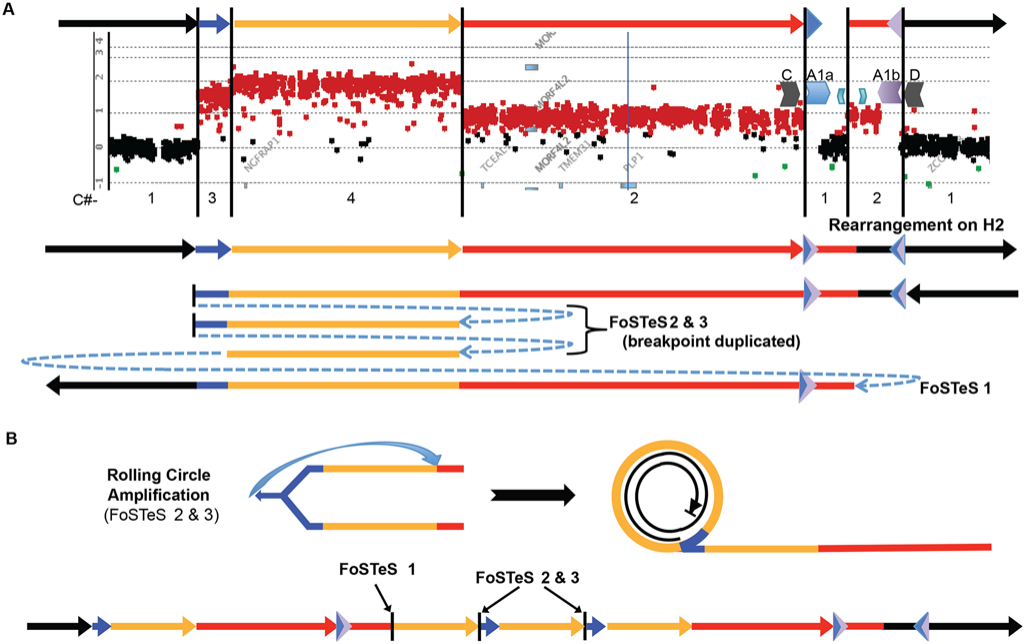
A quadruplication and potential rolling-circle mechanism of rearrangement. **A**) P113 aCGH result is shown. At top, colored arrows represent segments of triplicated (blue), quadruplicated (gold), and duplicated (red) sequence in this individual. A1a and A1b are represented by inverted blue and purple triangles, respectively. Copy number (C#) of the various segments is enumerated below the aCGH result, as is the rearrangement structure on the H2 haplotype (see S9 Fig). Breakpoints are depicted by blue dashed arrows in the schematic of the rearrangement and are labeled with the template switching events associated with the junction. Breakpoint sequences are depicted in S6 Fig, and dPCR data indicating duplication of FoSTeS events 2 & 3 is in S5 Table. **B**) The proposed rolling circle mechanism leading to the triplication and quadruplication is depicted, with the FoSTeS events 2 & 3 leading to invasion of the fork into the already replicated strand. This establishes the rolling circle, depicted on the right. The event ends with a double strand break or a fork disassociation event, and the breakpoint involving FoSTeS event 1 (S6 Fig). The structure of the overall rearrangement is depicted at the bottom, with colors relating to panel A for orientation.

We have discerned two junctions from patient P113 with proximal quadruplication and duplication of *PLP1* using long-range PCR (Figs. 5A, S6). Junction 1 consists of one fork stalling and template switching (FoSTeS) event—FoSTeS 1 (Fig. 5A). The second junction, between the proximal end of the triplication and the distal end of the quadruplication, consists of FoSTeS events 2 and 3 (S6 Fig for sequences of all junctions). We determined that the rearrangement was on the inverted H2 allele using PCR genotyping of the haplotype present in P113 (S9 Fig). Additionally, digital PCR (dPCR) data indicate that the FoSTeS 1 occurs in one copy, and FoSTeS 2/3 occurs in 2 copies (S5 Table).

This quadruplication rearrangement is also associated with a *de novo* point mutation (G insertion) ~50 bp away from the junction that appeared to occur concurrent with the rearrangement, as observed for other CGR mediated by a replicative process (S6 Fig) [20]. The mechanism by which copy number increased from 3 to 4 copies and generated the quadruplication is suggestive of a rolling circle amplification, wherein one breakpoint is repeated twice in the process of replicating ~280 kb (S5 Table) [22,36–38]. The FISH data for this individual shows the rearrangement to be contained on the X chromosome, and family data including the proband P113, his mother P154, his affected uncle P117, and grandmother P088 suggest that the structure is stable in 4 individuals from 3 generations, diminishing the likelihood of recombination-based amplification (Figs. 2D and S5). If the amplification were mediated by NAHR (see S10 Fig), this rearrangement would contain two templates for subsequent rounds of amplification. Therefore, the rearrangement in P113 should be twice as likely to undergo expansion as the proposed intermediate. Additionally, the presence of monomorphic SNPs throughout the region of copy number gains on SNP arrays indicates that the rearrangement was intra-chromosomal, as was found for the 13 patients with DUP-TRP/INV-DUP rearrangements (S11 Fig). The quadruplication-containing CGR was observed to transmit stably and co-segregate with disease through three generation (S5 and S11 Figs). A proposed mechanism for the rearrangement occurring in one complex quadruplication event is shown in Fig. 5 and consists of a rolling-circle amplification of the triplicated and quadruplicated segments.

## Discussion

*PLP1* is surrounded by LCRs of variable size and sequence similarity; previous studies have shown that such genomic architecture renders this region unstable and susceptible to rearrangements, leading to PMD [16,23,24]. We show that DUP-TRP/INV-DUP rearrangements are a frequent CGR product at the *PLP1* locus and that they are facilitated by a complex IR but specifically mediated via the ~20 kb A1a and A1b 99% identical repeats. These particular IRs are not only driving CGR observed in patients but additionally mediate a common SV polymorphism—copy-number neutral inversions at Xq22.2. The latter can complicate interpretation of CGRs in the region; a proposed breakpoint can also appear in an unaffected individual in the guise of an inverted allele [13]. Additionally, the recurrence of this inversion might confound the correlation of diagnostic SNPs with structural information, leading to the underestimation of the frequency in a population [39]. These data suggest that IRs with a high degree of identity that are involved in non-pathogenic inversions can also drive seemingly recurrent breakpoints in non-recurrent rearrangements associated with disease and that this occurs at multiple genomic loci [1,12,13]. Indeed, a previous determination of genes potentially subject to CNV via DUP-TRP/INV-DUP due to proximity of homologous IRs predicted the *PLP1* gene might be affected [2].

The proximal junctions, or Jct2, in the DUP-TRP/INV-DUP rearrangements at the *PLP1* locus are depicted in S6 Fig. Jct2 is non-recurrent in the 16 individuals, with different genomic coordinates for each breakpoint. Interestingly, investigation revealed that 14 of 16 Jct2 sequences contained microhomology of 1–4 bp at one or more of the FoSTeS events in the junction. Two of these Jct2 sequences involved larger stretches of microhomology; one contained an *Alu-Alu* chimeric event with 47 bp of perfect identity at the junction and the second CGR contained two *Alu-Alu* chimeras, one containing 14 bp and the other with 34 bp of perfect identity at the junction (Table 1). These data suggest that a replicative mechanism is involved in the formation of Jct2 in a majority of cases. Previously, we proposed that MMBIR or NHEJ could be responsible for Jct2 [1]. Here, we expand this ‘”two-step hypothesis” to include homeologous recombination within divergent repeats or similar sequences [33,40]. This is especially relevant to *Alu-Alu* mediated junctions, where the region of perfect identity may not be extensive enough to employ homology-driven repair, but extensive base-pairing outside the region of identity could aid in driving a recombination coupled replication driven rearrangement process at these loci [34]. In the 16 patients with DUP-TRP/INV-DUP rearrangements presented, 2 contain a Jct2 breakpoint resulting in the formation of a chimeric *Alu* element.

We hypothesize that the PMD-associated CGR are caused by BIR or MMBIR; these replicative processes have been shown to be error-prone, perhaps because they utilize a polymerase/replisome with reduced fidelity (induced point mutations) as well as reduced processivity (template switching) relative to intergenerational DNA polymerases [20,41]. Evidence now indicates that BIR/MMBIR-associated mutation results from conservative replication coupled with a migrating bubble [42,43]. Thus, DUP-TRP/INV-DUP CGRs involving *PLP1* have the potential to additionally impact patient health through point mutations on the X chromosome. These hypotheses need further investigation through large-scale genomic sequencing. Nevertheless, although few in number, *de novo* point mutations apparently acquired concomitantly with the DUP-TRP/INV-DUP rearrangement in P250 (insertion of an A) and the quadruplication rearrangement in P113 (insertion of a G) were not seen in the corresponding, contiguous (non-breakpoint containing) section of the X chromosome for these intrachromosomal events, a finding consistent with observations made at the *MECP2* locus and *de novo* mutation with CGR formation [20]. Given that on average, ~600 bp were sequenced at each junction, this suggests a rate of 2 mutations in ~15 kb of sequencing, consistent with the elevated point mutation rate observed in association with replication-based mechanisms of repair [20,41].

Junction 1 is present at seemingly identical loci, occurring within a complex inverted repeat structure in the 16 DUP-TRP/INV-DUP rearrangements studied. Further analysis has shown that at least one of these breakpoint junctions is in a region of 24 bp of microhomology and three occur within a >8 kb region of identity within A1a and A1b. The proposed mechanism for Jct1 is BIR within a region of ectopic, inverted homology [1]. Our data reveal that the template switch can occur within smaller regions of identity within A1a and A1b, suggesting that either MMBIR or homeologous recombination, rather than an homologous recombination within IRs may be an alternative mechanism for the formation of these seemingly recurrent junctions [22,31].

Previously, a study of 36 PMD patients identified 3 cases with duplicated copies of *PLP1* inserted outside of Xq22 [24]. Conversely, in this study all 16 subjects with junctions in IRs contain the extra copy or copies of the gene on Xq22, therefore suggesting that the mechanism of CNV results in a contiguous rearrangement (triplicated or duplicated regions in tandem, Fig. 2, Table 1). Additionally, all 16 individuals queried by Southern blotting and/or qPCR methodologies indicate that the A1a and A1b inverted LCRs mediate *PLP1* DUP-TRP/INV-DUP rearrangements. Although two other IRs in the region, albeit with less sequence identity (the 93% identical outer C/D and 87% identical innermost A2/A3 repeats), could presumably mediate the junction between distal duplication and distal triplication breakpoints, these 16 cases use the A1a and A1b specific repeats. A1a and A1b are ~20 kb in length (versus ~30 kb for C/D and ~10 kb for A2/A3) and are separated by ~50 kb (versus ~140 kb for C/D and ~30 kb for A2/A3). Therefore, the higher level of sequence identity between the A1a and A1b repeats (~99%), added to the shorter inter-repeat distance and the length of the LCR may both increase the likelihood of NAHR leading to the inversion [28,44] and potentiate these repeats as substrates for replication pausing, fork invasion, and reversal through BIR [31]. This is the second locus for which DUP-TRP/INV-DUP cases with recurrent Jct1 mediated by IRs has been described. In *MECP2* DUP-TRP/INV-DUP, the K1 and K2 LCRs participate in both non-pathogenic inversions and the rearrangements present in patients [1,27]. Such empirical studies may enable refinement of current predictions for IRs that can predispose regions of the genome to DUP-TRP/INV-DUP [2].

Our data further implicate a “two-step process” of BIR paired with MMBIR to generate CGRs resulting in duplication of copy number sensitive genes proximal to IRs [1]. The rearrangements in the 16 patients with DUP-TRP/INV-DUP contain just two junctions that result in four copy number transition states. This complex pattern on array CGH is due to just two template switches, Jct1 occurring within the LCRs A1a and A1b distal to the *PLP1* gene and resulting in an inversion, and Jct2 occurring at varying locations proximal to junction 1 and resuming the pattern of normal replication, resulting in a rescue from the potential formation of a dicentric chromosome (Fig. 4) [45].

The observations at the quadruplication-containing CGR in P113 are consistent with rolling-circle amplification (Fig. 5). The rarity of quadruplication at *PLP1* could be due to selective pressures from the increased severity of PMD with additional copies of *PLP1* (4 versus 3); it is notable that the quadruplication observed herein does not include the dosage sensitive *PLP1* gene [14]. One junction in this CGR (between IRs A1a and A1b) occurs at a similar location as in the DUP-TRP/INV-DUP structures, and PCR genotyping suggests that the interpretation of the rearrangement is complicated by the inversion structural variation, resulting in H2 (S9 Fig). At the proximal junction, the fork template switches twice, invading upstream and leading to a rolling-circle [22,36–38]. After almost two complete copies of the circle (35 kb short of the overall 280 kb), the next junction is a template switch from the proximal end of the quadruplicated region to the distal end of the duplicated region within the LCR region. Our observations are most parsimoniously explained by a rolling-circle amplification event, as predicted for higher-order genomic segment amplification in the MMBIR model [22]. Due to the observations of: i) triplicated and quadruplicated segments, ii) the accompanying point mutation associated with CGR formation, and iii) the prevalence of intrachromosomal rearrangements at this locus, a replicative model for CGR formation is likely [20,42,43]. The quadruplication-containing CGR provides evidence for an important next step in the MMBIR model, allowing for higher-order amplification to occur, as is often observed in cancer [22,46,47].

In summary, our studies confirmed a unique rearrangement product consisting of a DUP-TRP/INV-DUP structure in 16 individuals, with 6 containing triplication of *PLP1* [1]. We also elucidated a common, recurrent inversion polymorphism between two IRs distal to this gene. Jct1 occurs between the same repeats that mediate the non-pathogenic inversion, and sequencing of a DUP-TRP/INV-DUP breakpoint within the LCRs showed that these junctions can occur within short stretches of identity within a larger repeat of ~20 kb. This study of breakpoint junctions involved in both DUP-TRP/INV-DUP and higher-order amplification leading to quadruplication implicate replicative mechanisms in the generation of these CGRs. Additionally, we provide experimental evidence supporting the contentions that: i) IRs contribute to genome instability, ii) LCRs can mediate replication-based mechanisms, and iii) short repetitive sequences, such as *Alu*, can provide microhomology to facilitate template switching. The prevalence of DUP-TRP/INV-DUP events involving *PLP1* brings attention to the importance of this mechanism and the potentially broader impact of this rearrangement structure in gene and genome evolution.

## Materials and Methods

### Inversion Ascertainment

To determine whether there is a polymorphic inversion in the LCRs distal to *PLP1*, we examined the genomic information for 9 individuals contained in the human genome structural variation (HGSV) track of the UCSC Genome Browser [8,25,26]. The HGSV track (hg18) contains data on discordant fosmid end sequences from libraries of 9 individuals from diverse geographical regions. Discordant end sequence orientations of fosmids spanning LCRs A1a or A1b [23] and having both ends present in unique sequence (not LCRs) indicate potential inversions [26]. Individuals with at least one clone independently spanning each of the LCRs suggest that there is an inversion between the two repeats (S1 Fig).

### Inversion Haplotypes and Analysis

Phased data from the 1000 genomes project [29] was used to create plots of two haplotypes in the region spanning from LCRs A1a to A1b (Hg19 coordinates, ChrX:103223669–103324337) [48]. One thousand genomes data was cross-correlated with homozygous genotypes determined from Southern Blots to elucidate phased haplotypes that contain inversion alleles. Results were plotted using custom (in-house) scripts implemented in the R programming language (S4 Fig).

### Personal Genomes from Subjects and Patients Investigated

Families with PMD or rearrangements of Xq22.2 including *PLP1* were obtained by physician referral or self-referral. Patients were enrolled through informed consent in research protocols approved by the Institutional Review Boards at Baylor College of Medicine (BCM) and the Nemours Alfred I. duPont Hospital for Children. The rearrangements present in patients BAB1290, BAB1612/P374, BAB2389, P250, P255, P500, P518, and P558 were published previously [1,24,30]. Two of the patients with *PLP1* triplication (P518 and P674) were described as having more severe disease than patients with duplication [14]. Control DNAs from HapMap individuals [48] were obtained from the Coriell Institute for Medical Research cell repositories.

### DNA Digestion and Fragment Separation for Southern Blotting Analysis

Approximately 10 μg of genomic DNA from each patient was digested using BssSI. The DNA was diluted to 60 μl and digested for 4 hours at 37°C with 16U, heat inactivated at 80°C for 20 minutes, and the digest was then repeated with 12U for 3 hours and subsequent heat inactivation (leading to a 10-fold overdigestion). The digested DNAs were then precipitated and concentrated using standard sodium acetate precipitation, and were reconstituted in 25 μl of water with gentle mixing overnight. Concentrations were determined using a NanoDrop spectrophotometer, and samples were then loaded along with an 8–48 kb ladder on a 0.6% Tris- Boric Acid-EDTA (TBE) gel and run in 1X TBE buffer for ~3 days at 50–60 volts. DNA restriction digestion products, *i*.*e*. bands on gels, were then visualized with ethidium bromide staining.

### Probe Design for Southern Blot Analyses

Probe DNA was prepared using primers A1a proximal probe For- 5′-AATGCAGCTCAAAGGAAAGC-3′ and A1a proximal probe Rev- 5′-AGCCACTGACCAGTGATTTTC-3′ and amplifying a 514 bp fragment from BAC clone RP11–462K21 (https://bacpac.chori.org) DNA prepared using a QIAprep spin miniprep kit. The resultant PCR bands were resolved on 1% agarose and Tris-Acetate-EDTA gels and purified using a Zymoclean Gel DNA Recovery Kit (Zymo Research). Probe DNA was validated by Sanger sequencing, using both forward and reverse primers and was frozen at-20°C in 90 ng aliquots.

### Southern Blotting

Southern Blotting was carried out as previously described [23]. Briefly, DNAs were subjected to electrophoresis for sufficient duration to distinguish 25 and 29 kb fragment sizes and were then transferred to a Sure Blot positively charged nylon membrane by standard ‘sandwich’ methodology for 2–3 days. Approximately 80 ng of DNA was labeled with ^32^P-dCTP by random priming for 2–4 hours at 37°C using the Random Primed DNA labeling kit (Roche). Membranes were pre-hybridized for 4 hours in 10% dextran sulfate/1M NaCl/1%SDS (hybridization solution) with 4mg of sheared salmon sperm DNA at 65°C. Probe was pre-associated in hybridization solution with ~1mg sheared placental DNA at 65°C for ~2 hours, then added to the pre-hybridized membrane. Hybridization was carried out at 65°C overnight (~18 hours). The following day, the blot was washed and analyzed using autoradiography for bands corresponding to *PLP1* A1a structural haplotype information (~25 and 29 kb).

### Array CGH Analyses

To determine the size, genomic content, and extent of *PLP1* rearrangements, a high-density oligonucleotide array from Agilent was custom-designed to examine PMD patients. The 4 x 44 K microarray was designed using the Agilent eArray website (https://earray.chem.agilent.com/earray/) and was used to visualize the rearrangements of three patients in this study (BAB1290, BAB1612/P374, and BAB2389), the family containing individuals BAB3698, BAB3699, BAB3700, and BAB4179, and to complement existing array data for P500, P1407, and P113. The family of P113 was explored using Agilent arrays, including patients P113 and P117, as well as the mother of P113, P154, and grandmother, P088, who are both carriers. Probe labeling and hybridization were conducted as previously described, with NA15510 and NA10851 used as reference DNAs for female and male individuals, respectively (Accession GSE63594) [1]. Purified DNA samples from P113, P250, P500, P518, P558, P642, P674, P820, P842 and P1150 were submitted to NimbleGen for array service with normal male control NM002 as a reference. The NimbleGen X chromosome CGH fine-tiling array with oligonucleotide probes of 45 to 85 bases in length with median spacing of 106 bp throughout the whole X chromosome was used. Patient DNA samples P255/P298, BAB1612/P374, P1389 and P1407 were submitted to the Biomolecular Core Lab at duPont Hospital for Children for hybridization to Affymetrix Cytogenetics 2.7M Array. DNA sample P1407 was submitted to Coriell’s Genotyping and Microarray Center for hybridization on Affymetrix Genome Wide Human SNP Array 6.0. All data from Affymetrix arrays were analyzed with GeneChip Command Console Software AGCC. NimbleGen and Affymetrix Cytogenetics array data were aligned with qPCR data and plotted using the R programming language (Affymetrix and NimbleGen data are under Accession GSE64122) (Figs. 2B, C, D and S5).

### Semi-Quantitative Multiplex PCR (qPCR) for Detection of Copy Number

Semi-quantitative multiplex PCR was performed using a QIAGEN Multiplex PCR kit according to the manufacturer’s protocol to analyze regions on the X chromosome in and surrounding *PLP1* to determine copy number. Primer pairs were selected using the NCBI primer design tool (primers available upon request). In each experiment, five control DNAs were used, two known to have duplications in the region of interest without CGRs and three normal controls known to be single copy in the region of interest. A primer pair that amplifies a region of the human *dystrophin* (*DMD*) gene on the *p*-arm of the X chromosome was included in each multiplex reaction for amplification of a single-copy region. Products were separated by electrophoresis on a 4% NuSieve 3:1 agarose gel (Lonza, Walkersville MD) and stained with ethidium bromide. Net intensity of each band was determined using a Molecular Imaging system with Kodak Gel Logic Imaging software or AlphaImager HP. Copy number was determined by calculating the ratio of the net intensities of bands in the test region to *dystrophin* single-copy region for each DNA sample and then normalized by dividing by the average of the ratios of test region to *dystrophin* of the three normal controls. Theoretical ratios were: one, single-copy; two, duplication; three, triplication. Alternatively, quantitative PCR was performed as above except that one primer of each pair was labeled with 6-FAM and samples were submitted to the Biomolecular Core Lab at duPont Hospital for Children for capillary electrophoresis on an ABI PRISM 3130XL DNA Analyzer. Analysis of copy number was determined as above, by using area under the peak as determined by Peak Scanner software rather than net intensity. Triplicated, quadruplicated and duplicated regions were mapped to within several kb of their endpoints using these methods.

### Fluorescent *in situ* Hybridization (FISH) to Confirm Copy Number

Interphase nuclei and metaphase chromosomes were prepared from 700 μl of whole blood stored in sodium heparin Vacutainer tubes as follows. Blood was placed in α-MEM, 20%FBS, 1% L-glutamine, 50 μg/ml gentamycin and treated with 150 μl Phytohemagluttenin (Invitrogen, Carlsbad CA). The cultures were incubated at 37°C for 72 hours in upright position after which they were treated with 100 μl colcemid by trituration followed by incubation at 37°C for 30 min. Cultures were then subjected to centrifugation at 350 x g for 6 minutes. The supernatant liquid was discarded and the pellet was suspended in 10 ml 75mM KCl pre-warmed to 37°C and incubated at 37°C for 15 minutes. Then 1 ml of fixative (3:1 mixture of methanol:acetic acid) was added slowly. The preparation was washed 3 times in 10 ml of fixative with pelleting by centrifugation at 350xg for 6 minutes after washes. The resulting pellet of interphase and metaphase chromosomes was then stored in fixative at -20°C. Chromosomes and nuclei were dropped onto pre-cleaned Fisherbrand slides in a CDS-5 Glovebox environmental Chamber, (Thermotron, Holland Michigan) set at 25°C and 50% humidity. Slides were stored at -20°C in a vacuum under dessication until use.

FISH was performed using cosmid clone U125A1 and BAC clone RP13–188A5 obtained from the BACPAC resource center. Cosmid and BAC DNAs were isolated using the QIAGEN Plasmid purification and QIAGEN Large-Construct kits, respectively. One μg of U125A1 DNA was labeled with Biotin-16-dUTP and 1μg RP13–188A5 DNA was labeled with digoxigenin using the DIG-Nick Translation Mix. Labeled probes were purified using Nuctrap probe purification columns according to the manufacturer’s protocol. After hybridization to chromosomes and nuclei according to standard protocol, biotinylated U125A1 was bound to Cy3-labeled streptavidin, further amplified with biotinylated antiavidin (Vector Laboratories, Burlingame CA) and detected with a second layer of Cy3-labeled streptavidin. Simultaneously, RP13–188A5 labeled with digoxigenin was coupled with mouse antidigoxigenin, detected with rabbit anti-mouse FITC (Jackson ImmunoResearch Laboratories) and further amplified with goat anti-rabbit FITC antibody. Nuclei and chromosome spreads were counterstained with 4’,6-diamidino-2-phenylindole (DAPI) and cover slips were mounted using Vectashield antifade solution. Images were captured using a Leica DM RXA2 fluorescence microscope and Openlab imaging software (Perkin Elmer, Waltham MA).

### STR and SNP Analyses to Determine Origin of Extra Genomic Copies

To examine whether an intra- or inter-chromosomal origin occurred for the extra genomic segments in each patient’s genome, we analyzed 2 STRs and 9 single SNPs within the duplicated/triplicated region common to most patients. Sites were chosen based on marker genotypes displaying a high degree of heterozygosity in HapMap samples. S6 Table depicts the dbSNP identifiers, locations with respect to Chromosome X sequence NT_011651.17, and primers used to amplify the SNP or STR. Regions of interest were amplified from DNA with HotStar *Taq* DNA polymerase (Qiagen) for products <1kb or Expand High Fidelity PCR system (Roche) for products >1kb. Patients P250, P255/298, BAB1612/P374, P500, P518, P558, P642, P674, P820, P842, P1150, P1389, and P1407 were analyzed. Products containing the STR were amplified with one primer within the pair fluorescently-labeled so product sizes could be evaluated by capillary electrophoresis on ABI’s PRISM 3130 XL DNA Analyzer. Products containing the SNP were purified using QIAquick PCR or Gel purification kits, then sequenced with the Big Dye Terminator kit v. 3.1 (Life Technologies), according to the manufacturer’s instructions. Patients P113, P117 and carriers P154 and P088 were subjected to genotyping using the Illumina OmniExpress SNP array analyses at the human genome sequencing center of BCM. Data from the analyses was visualized by plotting the B allele frequencies versus the X chromosome coordinates encompassing the quadruplication genomic rearrangement, as well as the Log ratio of SNP intensity (S11 Fig).

### Jct2 Analyses

Inverse PCR was used to obtain the first junction of patient P1150. Briefly, DNA was digested with NheI and ligated to form circles. PCR primers were designed to amplify in opposite directions around the circle by long-range PCR using the Expand High Fidelity PCR system (S6 Table). When the PCR products were analyzed on an agarose gel, a product was found that was unique to the patient. The product was subjected to DNA sequencing according to the manufacturer’s instructions, then purified with the Filtration Cartridge (Edge Biosystems, Inc., Gaithersburg MD) and separated using an ABI PRISM 3130 xl Genetic Analyzer. DNA sequence was analyzed using Vector NTI sequence analysis software.

Proximal junctions were obtained for the personal genomes from the remaining triplication patients by long-range PCR using appropriately positioned primers at the endpoints of copy number changes (Table 1 and S6 Table) in 25 μl reactions with 50–100 ng of patient DNA using TaKaRa LA Taq or using the Expand High Fidelity PCR dNTPack kit according to the manufacturers’ instructions. PCR products were prepared for sequencing by using the standard ExoSAP-IT protocol (Affymetrix, Santa Clara CA) or by using the Qiagen PCR purification kit and DNA sequencing reactions were performed as indicated above using primers used in amplification or internal primers as indicated in S6 Table. Sequences were aligned to the human genome reference sequence, and breakpoints are depicted in S6 Fig. We had previously reported the sequence across the junction in P255 [24].

### Jct1 Analyses

PCR was conducted across Jct1 from DNAs prepared from patients with DUP-TRP/INV-DUP CGRs using a QIAGEN Multiplex PCR. Two control DNAs duplicated through this region and three control DNAs with a single copy at this locus were amplified in parallel. Along with a *dystrophin* primer pair (Hdys 23F-6FAM and Hdys 23 R) for a single copy region of the human *dystrophin* gene, we used primers pairs V362H12-F19–6FAM and V362H12-R19 (red arrows), and V362H12-F24–6FAM and V362H12-R24 (black arrows), and V362H12-F19–6FAM and V362H12-R24 (one red, one black arrow) (Figs. 3E, S8, S6 Table). Fluorescently labeled PCR products were diluted 1:100 in sterile HPLC water and subjected to capillary electrophoresis using an ABI PRISM 3130 XL DNA Analyzer. Copy number analysis was performed as previously described using the Peak Scanner software [24].

To subclone breakpoints in PMD DUP-TRP/INV-DUP patients, we amplified patient DNAs containing rearrangements (from BAB1612/P374, BAB2389, and BAB1290) with PCR primers that anneal within the A1a and A1b LCRs and uniquely flanking primers. This yielded four overlapping segments of the two LCRs (S6 Fig). These PCR products were then subjected to electrophoresis in crystal violet 0.8% agarose gels, purified using the SNAP purification kit from Invitrogen, and cloned into TOPO XL cloning vectors. Resultant clones for each of the four segments were screened by digestion and sequenced in their entirety. At least two clones for each region, obtained from independent PCR reactions, were screened for the breakpoint and the corresponding A1a or A1b region. Sequence analysis was conducted using the Lasergene 9 DNA analysis software suite.

### Copy Number Analysis of Junctions in the Quadruplication by dPCR

Copy number of junctions in the quadruplication patients and a carrier were determined by dPCR using QuantStudioTM 3D Digital PCR System (Life Technologies), according to the manufacturer’s instructions. Concentration of DNA was determined by QubitR dsDNA BR assay (Life Technologies) using the Qubit 2.0 fluorometer (Life Technologies). Sample DNA was digested with SphI (NEBiolabs) to separate multiple copies of interest that may be located on the same molecule without disrupting the region of amplification. Digests were performed using 400 ng of DNA in a 10 μl reaction containing 10U of SphI and incubating at 37°C for 1.5 hr, followed by heat-inactivation of the enzyme at 65°C for 20 min. The digest was diluted to 40 μl with RNase-free water to yield a concentration of 10 ng/μl DNA. Primers and probes used in the dPCR assays are in S6 Table. Reactions for dPCR included 1x QuantStudioTM 3D Digital PCR Master Mix, 1x TaqMan Copy Number Reference Assay for human RNaseP (Life Technologies, Cat. # 4403328, VIC label), 1–1.5x PrimeTime qPCR 5’ nuclease assay (IDT, FAM label) for jct1 or jct2/3 and 40–60ng DNA in a 16 μl volume. Fifteen μl of this mix was used to load the Digital PCR 20K Chip (Life Technologies); chips were processed according to the manufacturer’s instructions.

## Supporting Information

S1 Fig Inversions in the HGSV. The Human Genome Structural Variation track (NCBI36/hg18) prediction of inversion from the UCSC genome browser. The locations of A1a and A1b are noted. Green horizontal lines indicate inversion fosmids (discordant end orientations) with respect to the reference sequence. Five individuals have strong support (both LCRs spanned) for an inversion—G248, ABC10, ABC11, ABC12, and ABC13, and three more have weak support—ABC7, ABC8, and ABC14; ABC9 appears to be homozygous for the reference orientation.
(PDF)

S2 Fig Breakpoint of the inversion in G248. One inversion fosmid spanning A1a from individual NA15510 (library G248) was previously sequenced in its entirety (GI:121495926)(Kidd et al. 2010). The other breakpoint of this inversion was not investigated, as the fosmid sequence was unavailable. Analysis of this clone with respect to the reference sequence for LCRs A1a (in blue) and A1b (in purple) revealed an apparent switch from paralogous sequence variants (PSVs) belonging to one LCR to those of the other. This occurred after 18525 bp of A1a in a block of 334 base pairs of identical sequence and prior to 1492 bp of A1b (Lindsay et al. 2006). Seven LCR A1a-specific PSVs in the 1000 bp proximal and 10 A1b-specific PSVs in 200 bp distal to the 334 bp region of perfect identity signified a historical recombination event between A1a and A1b. These data indicate a putative NAHR-mediated switch ~1500 bp from the end of LCR A1a, mediating the inversion. The breakpoint sequence occurred within 334bp of 100% identity (ChrX:103242193–103242526 in A1a) between the two reference LCRs at this location, and two putative PRDM9 binding sites/homologous recombination hotspot motifs are present at the distal end of the region of homology (sequence depicted in red)(Myers et al. 2008; Myers et al. 2010). These data implicate NAHR as the likely mechanism for the inversion.
(PDF)

S3 Fig Additional genotyping of inversion allele in phenotypically normal individuals. Ten individuals from the HapMap population studied for inversion. Gender of the individual is indicated by circles (female) or squares (male) above the figure. DNA identifiers (NA numbers) are consistent with Coriell names (http://ccr.coriell.org/), and fosmid libraries (ABC library identifiers) are as in Kidd et al. (Kidd et al. 2010). The population of origin for each individual and the genotypes are indicated at the bottom of the blot. H = Han Chinese, Y = Yoruban, C = CEPH, and U = unknown. Samples repeated between this blot and the one in Fig. 1 are indicated with an asterisk (*). Lanes 2 and 3 and lanes 7 and 8 were separated by samples that did not produce readily visible bands and were therefore removed from the figure. These lanes are indicated with white space in the image of the Southern blot. Quantitation of the blot is presented in S1 Table.
(PDF)

S4 Fig Inversions between A1a and A1b LCRs appear to be recurrent. Reference and inversion (H1 and H2) structural haplotypes inferred from Southern blotting were plotted over the sequence haplotypes from the 1000 genomes project. Individuals are denoted on the left—each female has two alleles (A1 and A2) whereas males only have one. Homozygous H2 alleles are highlighted in yellow. Each colored dot represents a non-reference sequence of a SNP at that given location. Red denotes a cytosine, blue an adenine, green is guanine, and gold is thymine, whereas a reference call is shown in white. The orange bars denote location of A1a and A1b repeats with inverted arrows indicating orientation. NA18947 is homozygous for H2, and A1 lies in the upper haplotype. The 6 other alleles from homozygous or hemizygous H2 individuals are on the bottom haplotype.
(PDF)

S5 Fig Array and semi-quantitative PCR results. **A**) Semi-quantitative PCR results are displayed below NimbleGen and Affymetrix array data. Location of *PLP1* denoted by vertical grey arrow, and array results are depicted as in Fig. 2, with duplications in red and triplications in blue. The position of the LCR region is shown in yellow (C and D repeats) and purple (A1a, A1b, A2 and A3 repeats). **B**) Agilent aCGH data is depicted, with red probes indicating amplification and green deletion. Shading in BAB3698 pedigree denotes location of aberration. Pedigrees for families of probands BAB3698 and P113 are shown. Details regarding arrays and PCR are discussed in the Materials and Methods section.
(PDF)

S6 Fig Jct2 and other breakpoint junctions from patients in the study. For DUP-TRP/INV-DUP cases, Jct2 is detailed with Dist. (-) representing the distal (minus strand) and the triplication start sequence; Prox. is the proximal region (plus strand) and the duplication start sequence. Mid. (where applicable) represents the middle, inserted sequence. BPT is the sequence of Jct2 itself. For other breakpoints junctions (P113, both junctions; P1150 ‘deletion’), proximal and distal are centromeric and telomeric, respectively. The vertical lines in the alignments indicate sequences that align perfectly. Red sequences indicate microhomology, and green sequence for BAB1290 indicates potential sequence involved in the second template switch. Jct2 of P518 involves two *Alu* mediated template switches 488 bp apart. The schematic below the P518 junction sequence depicts the rearrangement. P113 involves a complex rearrangement leading to quadruplication, as indicated in the text. The sequences for the two junctions in this individual are depicted, including the *de novo* single nucleotide addition found at the proximal side of the breakpoint. FoSTeS 1 occurred on an H2 haplotype, therefore there is no inversion at this breakpoint junction.
(PDF)

S7 Fig qPCR results for Jct1. Data showing the inversion Jct1 in patients with DUP-TRP/INV-DUP. DNA from patients with duplication and triplication and normal controls were amplified using primer pairs shown in Fig. 3E and S6 Table. The qPCR primer pairs amplify a unique region outside of the A1a LCR (in red), inside of both A1a and A1b LCRs (in black) or from the A1a LCR to a unique region outside (red/black pair below). These will give rise to one copy (red pair and red/black pair) or two copies (black pair) in a non-rearranged X chromosome in a male individual. DUP-TRP/INV-DUP (on right) will give rise to four copies amplified by the black pair (2x normal control) and three copies by the red pair and red/black pair (3X normal control). A duplication-containing individual will contain four of the black primer pair reaction (2x control) and 2 copies of both the red pair and red/black pair (2x control). These data are reflected in the graph above.
(PDF)

S8 Fig Jct1 cloning and sequencing. To clone the breakpoints from individuals (BAB1612/P374, BAB1290, and BAB2389) with DUP-TRP/INV-DUP rearrangements, the ~20 Kb LCR regions were PCR amplified in two overlapping fragments (relevant inversion allele shown). Black PCR primers anneal to either LCR A1a (blue segments, shortened to “A”) or LCR A1b (purple segments, shortened to “B”), whereas colored primers are unique to the flanking sequences of the LCRs. Red vertical line indicates location of Jct1. Multiple resultant PCR fragments for each region were then cloned and sequenced. Analysis showed varying degrees of homology between the two LCRs in different individuals, and succeeded in cloning the breakpoint from BAB1612/P374.
(PDF)

S9 Fig PCR genotyping of H2 haplotype in P113. Genotyping of H1 or H2 in HapMap patients NA10851 and NA18942 support Southern blot data (Figs. 1 and S3). Therefore, this PCR genotyping scheme was applied to P113 and resulted in the discovery of the H2 haplotype in this patient. The haplotype information simplifies the number of breakpoint junctions present in this individual from 3 to 2, as is shown in Fig. 5. The genotyping was done using PSVs present between A1a (A) and A1b (B) LCRs with TaKaRa GXL polymerase. The primers were: AFor 5’-AAGTCTCATTTAGTATTACGACTTACAATTCC-3’ BFor 5’- AAAGTCTCATTTAGTATTACAGCTTACAATTCT-3’ ARev 5’- GCGACTAACGTTGGATAGTCCT-3’ BRev 5’- ATGTGACCAATGTTGGATAGTGTC-3’ Therefore, AA amplifies an A1a LCR, BB an A1b, and AB amplifies an A1a to A1b LCR, and BA an A1b to A1a LCR.
(PDF)

S10 Fig Alternative, recombination-mediated two-step mechanism for quadruplication. Depicted is an alternative mechanism that could underlie the rearrangement found in P113. This recombination-mediated rearrangement would require two steps in separate generations to generate the complex rearrangement seen in the patient.
(PDF)

S11 Fig SNP array results for quadruplication. **A**) B-allele frequencies and **B**) log ratio of SNP genotyping results for P113 pedigree. Colors for dots indicate duplicated (red) triplicated (blue) and quadruplicated (orange) region.
(PDF)

S1 Table Semi-quantitative analysis of HapMap Southern blots. Southern blot genotyping of the inversion in 17 HapMap individuals was quantitated using GelAnalyzer 2010 software. Briefly, lanes and bands were designated according to the histograms, and normalization was determined automatically by the valley-to-valley normalization function, Resultant band intensities (Raw Volume) were used to generate ratios of the bands if more than one was present. This ratio is represented in the final column of the table, and heterozygous HapMap females have an expected ratio of 1. Asterisks indicate DNAs that were on both blots.
(PDF)

S2 Table Population distribution of H1 and H2. Southern blot genotyping of the inversion in 4 HapMap populations, as well as one individual of unknown population of origin.
(PDF)

S3 Table Results of SNP and STR genotyping. (PDF)

S4 Table Semi-quantitative analysis of patient Southern blots. Southern blot genotyping of DUP-TRP/INV-DUP patients was quantitated using GelAnalyzer 2010 software, as in S1 Table. This ratio is represented in the final column of the table. Ratios of ~2 in the patients indicate H2 rearrangements, while less than one indicate H1 (expected ratio of ~0.5). Carrier females from BAB3698 family and females without rearrangements have expected ratios of ~1.
(PDF)

S5 Table dPCR results for FoSTeS Jct 1 and 2/3 from qadruplication. The table depicts digital PCR results from Jct/FoSTeS 1 and Jct/FoSTeS 2/3 in patient P113, the affected uncle of the proband P117, and the carrier mother of P113, P154. In each experiment, FoSTeS 1 has a copy number of 1, and FoSTeS 2/3 has a copy number of 2. Pooled normal control individuals (6NLs) have neither junction. *Copies/genome is calculated for each junction as (copies/microliter jct)/([copies/microliter RNaseP]/2). Note that RNase P is autosomal and so has two copies per male or female genome (one on each allele). The junctions are located on the X chromosome; there is only one X allele in males, and it is affected in the patients. Female carriers have junctions on one X allele but not the other.
(PDF)

S6 Table Primers and probes used in the manuscript. SNP primers amplify *de novo* nucleotide changes, and Inv. PCR primers were used in inverse PCR of sample P1150. Additional primers are available upon request.
(PDF)
