# Supplementary figures and images for: Complex Genomic Rearrangements at the PLP1 Locus Include Triplication and Quadruplication
Source: PLoS Genet. 2015 Mar 6;11(3):e1005050. doi: 10.1371/journal.pgen.1005050 (PMC4352052; doi:10.1371/journal.pgen.1005050)

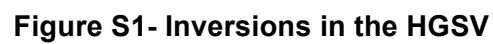

Supplement: S1 Fig — The Human Genome Structural Variation track (NCBI36/hg18) prediction of inversion from the UCSC genome browser. The locations of A1a and A1b are noted. Green horizontal lines indicate inversion fosmids (discordant end orientations) with respect to the reference sequence. Five individuals have strong support (both LCRs spanned) for an inversion—G248, ABC10, ABC11, ABC12, and ABC13, and three more have weak support—ABC7, ABC8, and ABC14; ABC9 appears to be homozygous for the reference orientation. (PDF) [file pgen.1005050.s001.pdf]

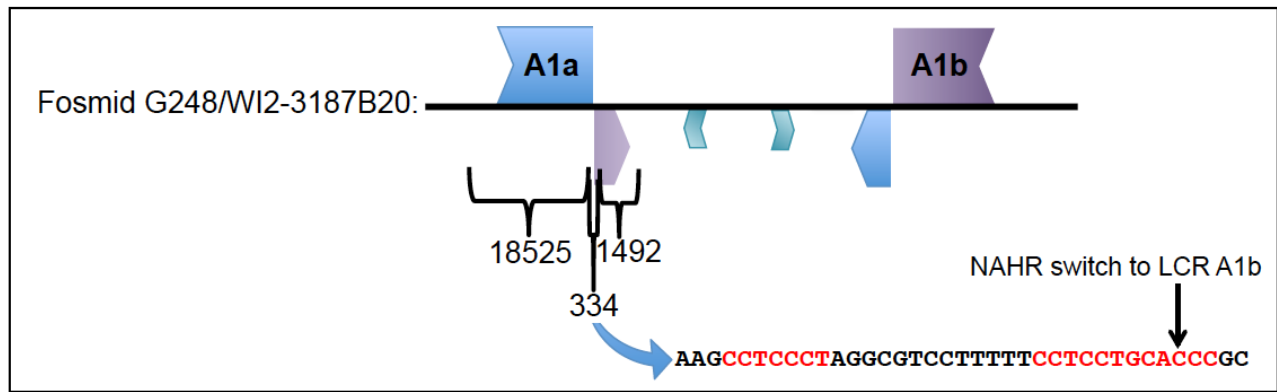

**Figure S2- Breakpoint of the Inversion in G248**

Supplement: S2 Fig — One inversion fosmid spanning A1a from individual NA15510 (library G248) was previously sequenced in its entirety (GI:121495926)(Kidd et al. 2010). The other breakpoint of this inversion was not investigated, as the fosmid sequence was unavailable. Analysis of this clone with respect to the reference sequence for LCRs A1a (in blue) and A1b (in purple) revealed an apparent switch from paralogous sequence variants (PSVs) belonging to one LCR to those of the other. This occurred after 18525 bp of A1a in a block of 334 base pairs of identical sequence and prior to 1492 bp of A1b (Lindsay et al. 2006). Seven LCR A1a-specific PSVs in the 1000 bp proximal and 10 A1b-specific PSVs in 200 bp distal to the 334 bp region of perfect identity signified a historical recombination event between A1a and A1b. These data indicate a putative NAHR-mediated switch ~1500 bp from the end of LCR A1a, mediating the inversion. The breakpoint sequence occurred within 334bp of 100% identity (ChrX:103242193–103242526 in A1a) between the two reference LCRs at this location, and two putative PRDM9 binding sites/homologous recombination hotspot motifs are present at the distal end of the region of homology (sequence depicted in red)(Myers et al. 2008; Myers et al. 2010). These data implicate NAHR as the likely mechanism for the inversion. (PDF) [file pgen.1005050.s002.pdf]

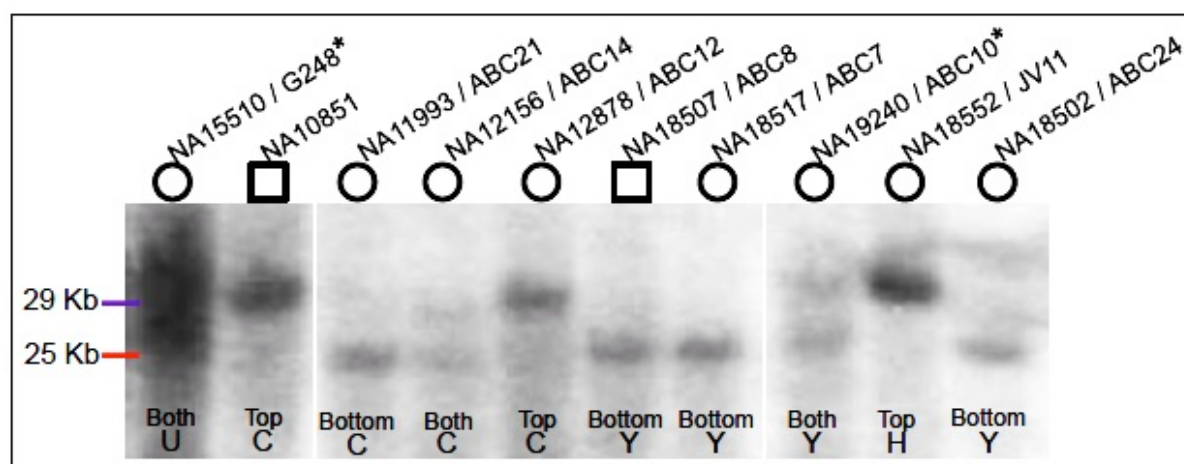

**Figure S3- Additional Genotyping of Inversion Allele in Phenotypically Normal Individuals**

Supplement: S3 Fig — Ten individuals from the HapMap population studied for inversion. Gender of the individual is indicated by circles (female) or squares (male) above the figure. DNA identifiers (NA numbers) are consistent with Coriell names (http://ccr.coriell.org/), and fosmid libraries (ABC library identifiers) are as in Kidd et al. (Kidd et al. 2010). The population of origin for each individual and the genotypes are indicated at the bottom of the blot. H = Han Chinese, Y = Yoruban, C = CEPH, and U = unknown. Samples repeated between this blot and the one in Fig. 1 are indicated with an asterisk (*). Lanes 2 and 3 and lanes 7 and 8 were separated by samples that did not produce readily visible bands and were therefore removed from the figure. These lanes are indicated with white space in the image of the Southern blot. Quantitation of the blot is presented in S1 Table. (PDF) [file pgen.1005050.s003.pdf]

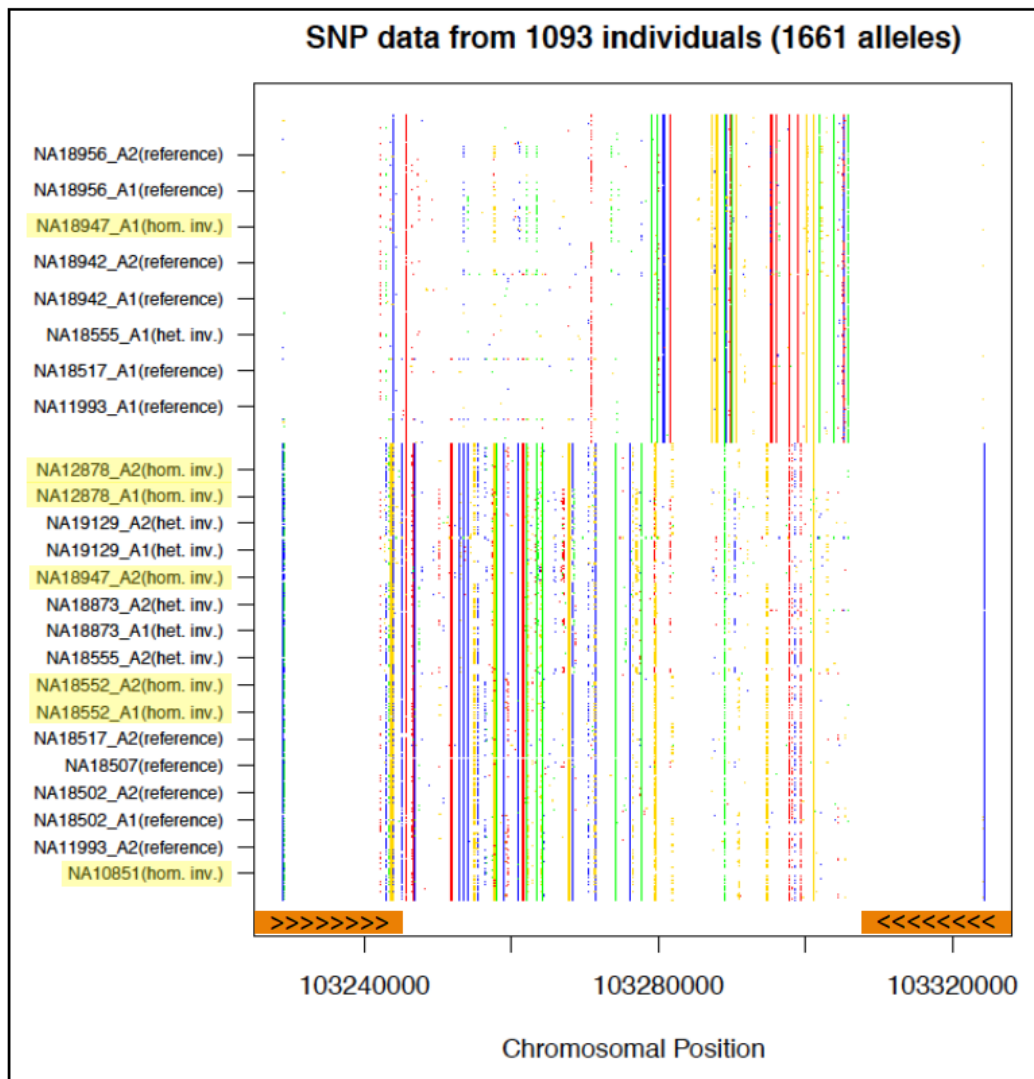

**Figure S4- Inversions Between A1a and A1b LCRs Appear to be Recurrent**

Supplement: S4 Fig — Reference and inversion (H1 and H2) structural haplotypes inferred from Southern blotting were plotted over the sequence haplotypes from the 1000 genomes project. Individuals are denoted on the left—each female has two alleles (A1 and A2) whereas males only have one. Homozygous H2 alleles are highlighted in yellow. Each colored dot represents a non-reference sequence of a SNP at that given location. Red denotes a cytosine, blue an adenine, green is guanine, and gold is thymine, whereas a reference call is shown in white. The orange bars denote location of A1a and A1b repeats with inverted arrows indicating orientation. NA18947 is homozygous for H2, and A1 lies in the upper haplotype. The 6 other alleles from homozygous or hemizygous H2 individuals are on the bottom haplotype. (PDF) [file pgen.1005050.s004.pdf]

Figure S5

A

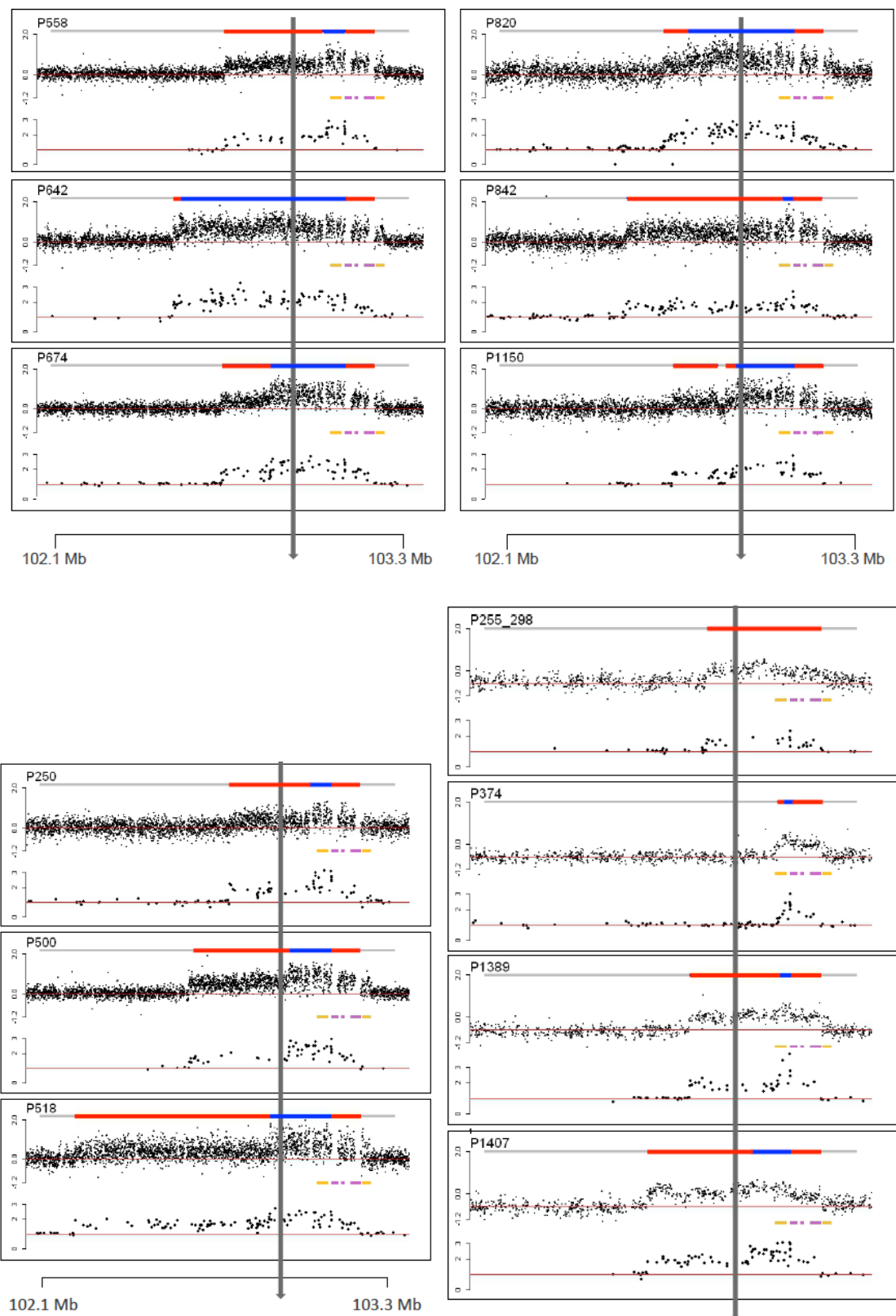

B

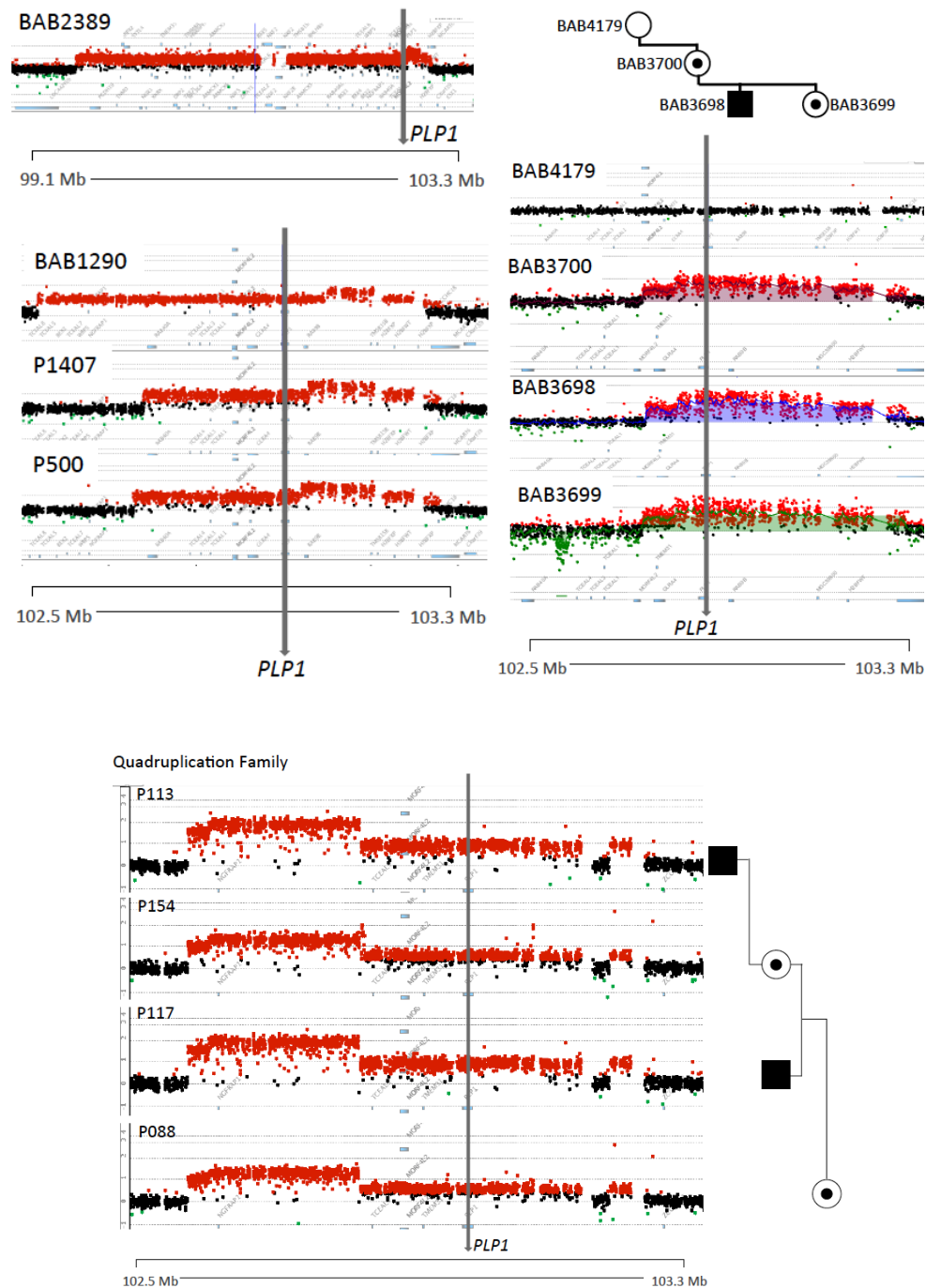

Figure S5- Array and Semi-Quantitative PCR Results

Supplement: S5 Fig — A) Semi-quantitative PCR results are displayed below NimbleGen and Affymetrix array data. Location of PLP1 denoted by vertical grey arrow, and array results are depicted as in Fig. 2, with duplications in red and triplications in blue. The position of the LCR region is shown in yellow (C and D repeats) and purple (A1a, A1b, A2 and A3 repeats). B) Agilent aCGH data is depicted, with red probes indicating amplification and green deletion. Shading in BAB3698 pedigree denotes location of aberration. Pedigrees for families of probands BAB3698 and P113 are shown. Details regarding arrays and PCR are discussed in the Materials and Methods section. (PDF) [file pgen.1005050.s005.pdf]

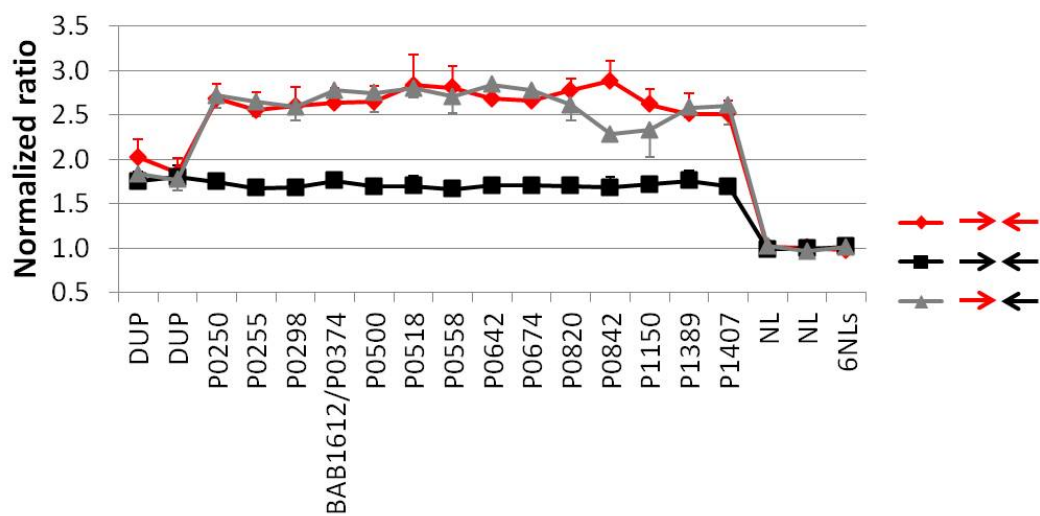

Figure S7- qPCR Results for Jct1

Supplement: S7 Fig — Data showing the inversion Jct1 in patients with DUP-TRP/INV-DUP. DNA from patients with duplication and triplication and normal controls were amplified using primer pairs shown in Fig. 3E and S6 Table. The qPCR primer pairs amplify a unique region outside of the A1a LCR (in red), inside of both A1a and A1b LCRs (in black) or from the A1a LCR to a unique region outside (red/black pair below). These will give rise to one copy (red pair and red/black pair) or two copies (black pair) in a non-rearranged X chromosome in a male individual. DUP-TRP/INV-DUP (on right) will give rise to four copies amplified by the black pair (2x normal control) and three copies by the red pair and red/black pair (3X normal control). A duplication-containing individual will contain four of the black primer pair reaction (2x control) and 2 copies of both the red pair and red/black pair (2x control). These data are reflected in the graph above. (PDF) [file pgen.1005050.s007.pdf]

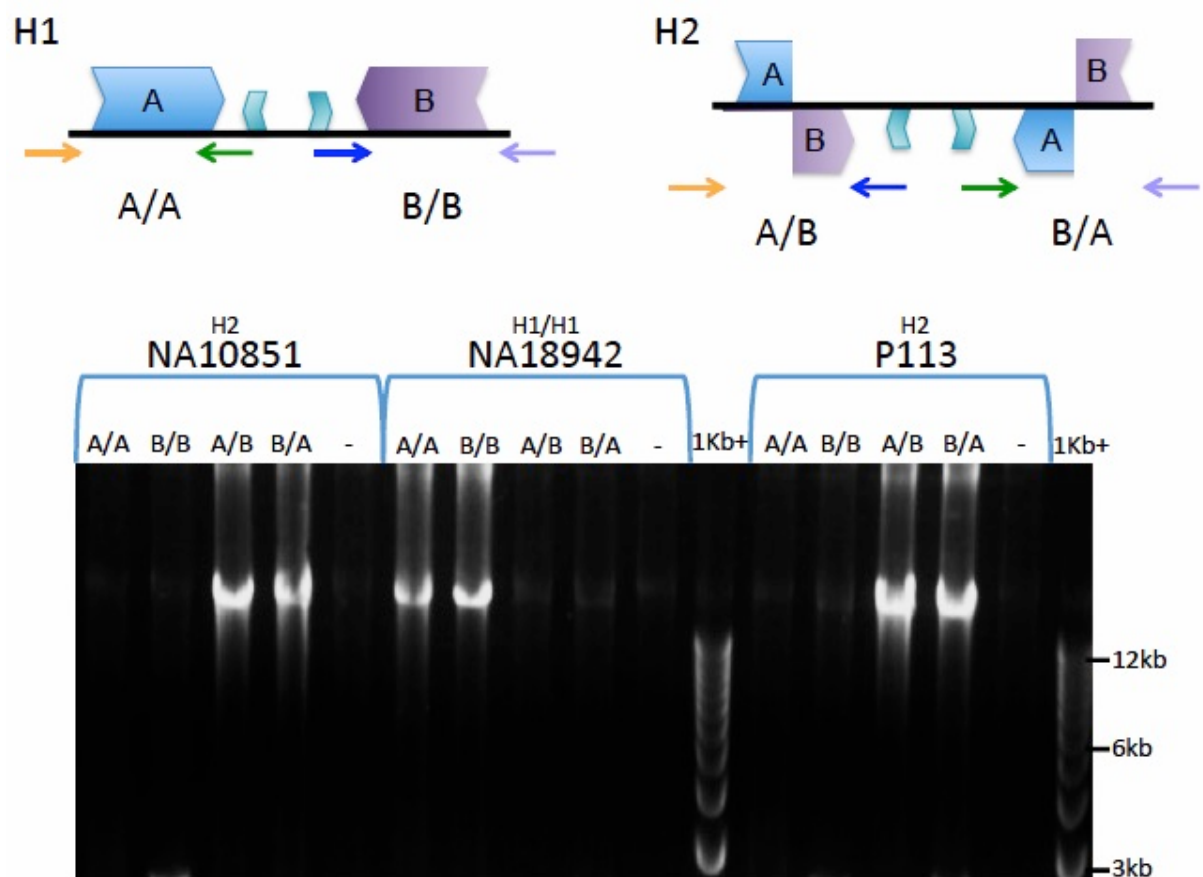

Figure S9- PCR Genotyping of H2 Haplotype in P113

Supplement: S9 Fig — Genotyping of H1 or H2 in HapMap patients NA10851 and NA18942 support Southern blot data (Figs. 1 and S3). Therefore, this PCR genotyping scheme was applied to P113 and resulted in the discovery of the H2 haplotype in this patient. The haplotype information simplifies the number of breakpoint junctions present in this individual from 3 to 2, as is shown in Fig. 5. The genotyping was done using PSVs present between A1a (A) and A1b (B) LCRs with TaKaRa GXL polymerase. The primers were: AFor 5’-AAGTCTCATTTAGTATTACGACTTACAATTCC-3’ BFor 5’- AAAGTCTCATTTAGTATTACAGCTTACAATTCT-3’ ARev 5’- GCGACTAACGTTGGATAGTCCT-3’ BRev 5’- ATGTGACCAATGTTGGATAGTGTC-3’ Therefore, AA amplifies an A1a LCR, BB an A1b, and AB amplifies an A1a to A1b LCR, and BA an A1b to A1a LCR. (PDF) [file pgen.1005050.s009.pdf]

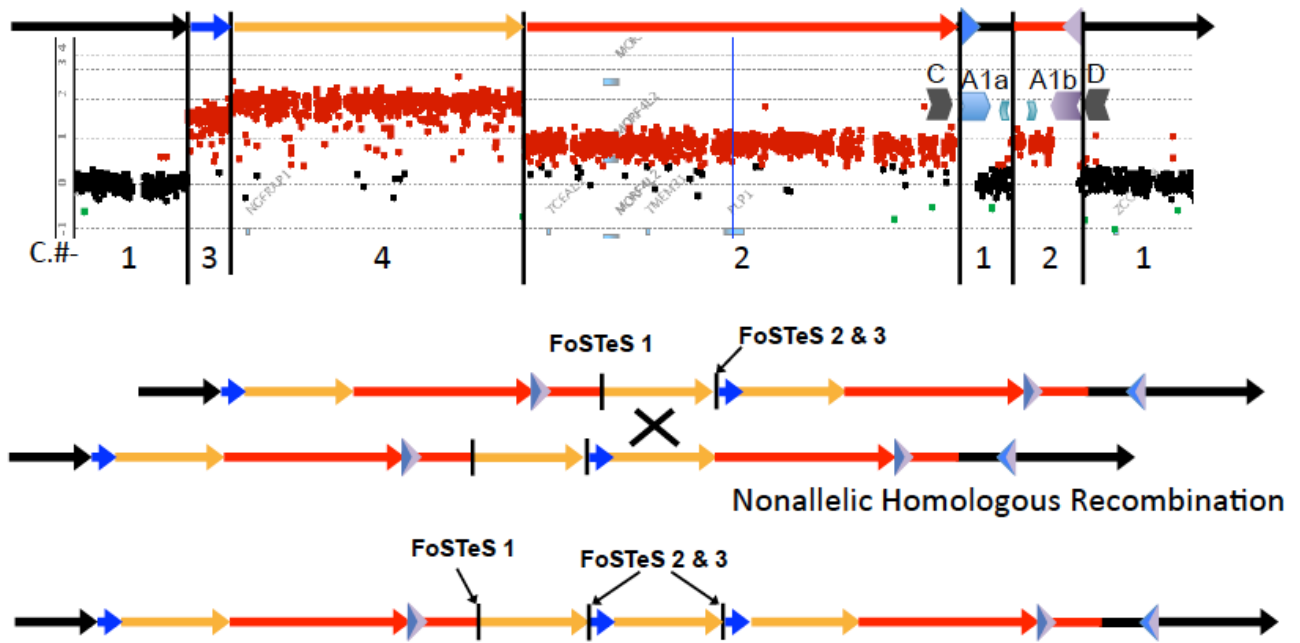

Figure S10- Alternative, Recombination-Mediated Two-Step Mechanism for Quadruplication

Supplement: S10 Fig — Depicted is an alternative mechanism that could underlie the rearrangement found in P113. This recombination-mediated rearrangement would require two steps in separate generations to generate the complex rearrangement seen in the patient. (PDF) [file pgen.1005050.s010.pdf]

**A**

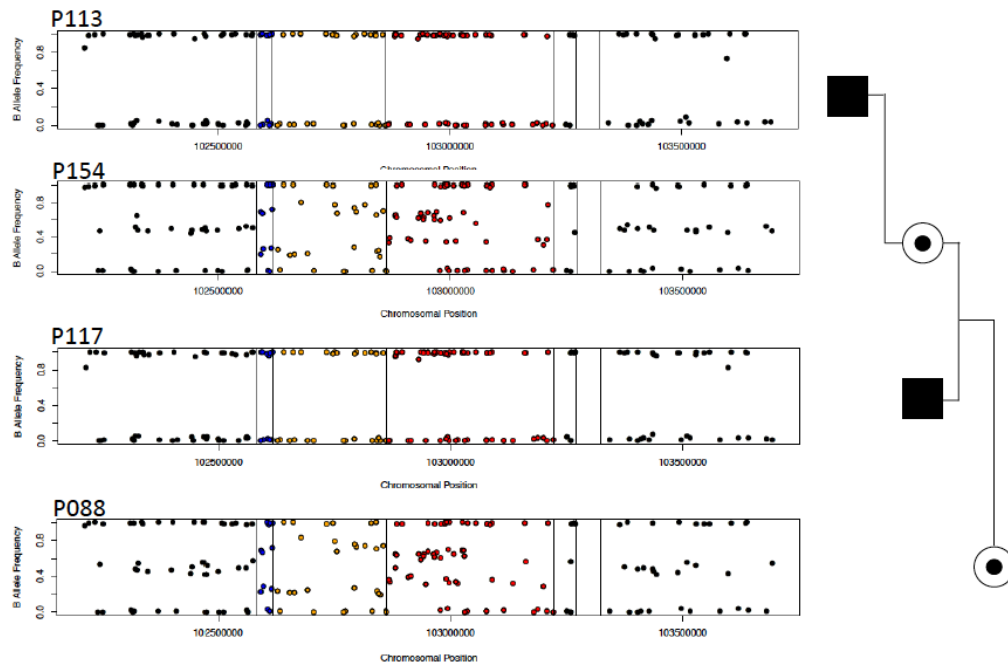

**B**

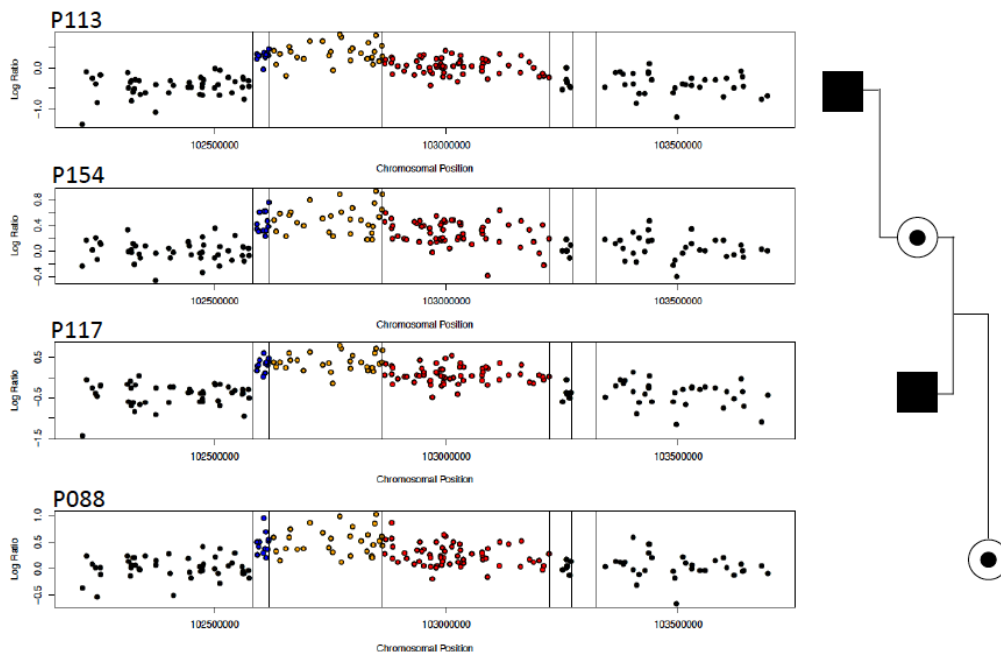

**Figure S11- SNP Array Results for Quadruplication**

Supplement: S11 Fig — A) B-allele frequencies and B) log ratio of SNP genotyping results for P113 pedigree. Colors for dots indicate duplicated (red) triplicated (blue) and quadruplicated (orange) region. (PDF) [file pgen.1005050.s011.pdf]
