# Supplementary material for: Complex Genomic Rearrangements at the PLP1 Locus Include Triplication and Quadruplication
Source: PLoS Genet. 2015 Mar 6;11(3):e1005050. doi: 10.1371/journal.pgen.1005050 (PMC4352052; doi:10.1371/journal.pgen.1005050)
Supplement: S6 Fig — For DUP-TRP/INV-DUP cases, Jct2 is detailed with Dist. (-) representing the distal (minus strand) and the triplication start sequence; Prox. is the proximal region (plus strand) and the duplication start sequence. Mid. (where applicable) represents the middle, inserted sequence. BPT is the sequence of Jct2 itself. For other breakpoints junctions (P113, both junctions; P1150 ‘deletion’), proximal and distal are centromeric and telomeric, respectively. The vertical lines in the alignments indicate sequences that align perfectly. Red sequences indicate microhomology, and green sequence for BAB1290 indicates potential sequence involved in the second template switch. Jct2 of P518 involves two Alu mediated template switches 488 bp apart. The schematic below the P518 junction sequence depicts the rearrangement. P113 involves a complex rearrangement leading to quadruplication, as indicated in the text. The sequences for the two junctions in this individual are depicted, including the de novo single nucleotide addition found at the proximal side of the breakpoint. FoSTeS 1 occurred on an H2 haplotype, therefore there is no inversion at this breakpoint junction. (PDF) [file pgen.1005050.s006.pdf]

Figure S6

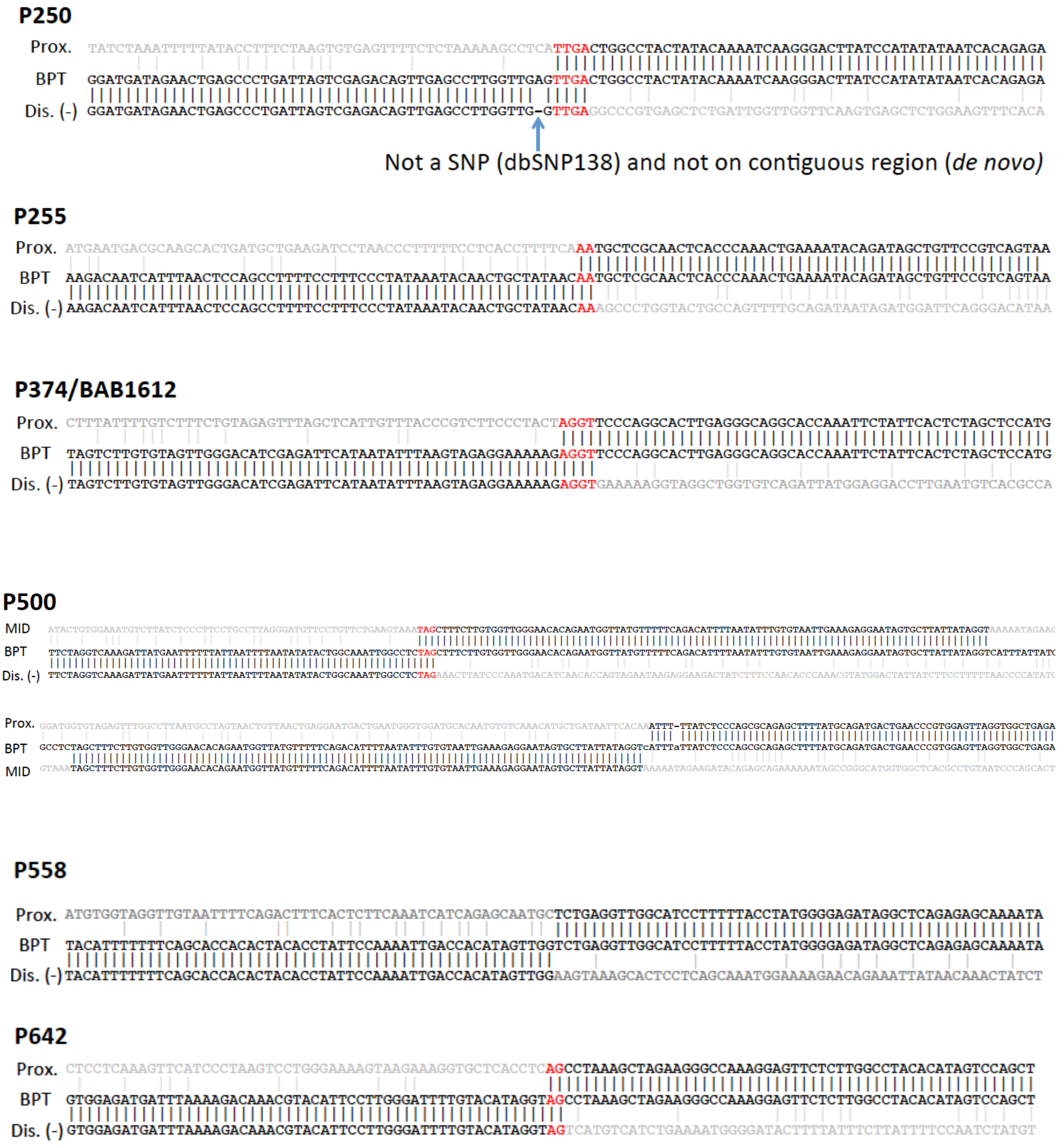
P250
Prox.
BPT
Dis. (-)
Not a SNP (dbSNP138) and not on contiguous region (de novo)
P255
Prox.
BPT
Dis. (-)
P374/BAB1612
Prox.
BPT
Dis. (-)
P500
MID
BPT
Dis. (-)
Prox.
BPT
MID
P558
Prox.
BPT
Dis. (-)
P642
Prox.
BPT
Dis. (-)

P518- Alu Sq2 to Alu Sg

488 bp

Distal bpt- 103004306 (-) /Middle section is 103003940 to 103004427 (+)/ Prox. is 102324702 (+)

Distal- (+) AluSq2

```
Dis. (-)  TTGGCCAGGCTGGTCTCAAACTCCTGACCTCAGGTGATCCACCCGCCTTGGCCTCCCAAAGTGCTGGGATTAC-AGGCGTGAGCCACTGCACCCGGCCAGATATGCACTCTTGTGTGTTTGATTT
BPT       TTGGCCAGGCTGGTCTCAAACTCCTGACCTCAGGTGATCCACCCGCCTTGGCCTCCCAAAGTGCTGGGATTACTGGGCGTGAGCCACCATGCCTGGCCAACATTTCTATATCCCCCTAGAACTAC
Mid. (+)  CATGTTGGTCAGGCTGGTTTCGAACTCCTGACCTCGTGATCCGCCGCTCGG-CTCCCAAAGTGCTGGGATTAC-AGGCGTGAGCCACCATGCCTGGCCAACATTTCTATATCCCCCTAGAACTAC
```

Middle- (-) AluSx3

```
Prox.(+)  AAATAGCAAGAGAACTAGAATTAGAAGTAGAGCCTGGGCCGGGCACAGTGGCTTACACCTGTAATCCCAGCACTTCGGGAGGCTGAGGCGGGCAGATCAC--GAGGTCAGGAGTTTGAGACCAG
BPT       CATTTATGTAAATCAAACACACAAGAGTGCATATCTGGCCGGGTGCAGTGGCTCACGCCTGTAATCCCAGCACTTTGGGAGGCCAAGGCGGGTGGATCACCTGAGGTCAGGAGTTTGAGACCAG
Mid. (+)  CATTTATGTAAATCAAACACACAAGAGTGCATATCTGGCCGGGTGCAGTGGCTCACGCCTGTAATCCCAGCACTTTGGGAGGCCAAGGCGGGTGGATCACCTGAGGTCAGGAGTTTGAGACCAG
```

Proximal- (+) AluSg

```
Prox. (+) AATGACCAAGATGGTGAAACCAAGTCTCTACTAAAAATACAAAAA-TTAGCCAGGCATGGTGGCGCGTGCCTGTAATCCCAGCTATCCAGGAGGCTGAGGCAGGAGAATCACTTGAACCCGGGA
BPT       CCTGGCCAACATGGTGAAACCCTGTCTCTATTAAAAATACAAAAA-TTAGCCAGGCATGGTGGCGCGTGCCTGTAATCCCAGCTATCCAGGAGGCTGAGGCAGGAGAATCACTTGAACCCAGGA
Mid. (+)  CCTGGCCAACATGGTGAAACCCTGTCTCTATTAAAAATACAAAAAATTAGCCATGCATGGTGGTGGATGCCTGTAATCCCAGCTACTCTGGAGGCTAAGACAGGAGAATCGCTTGAACCTGGGA
```

Middle- (+) AluSq2

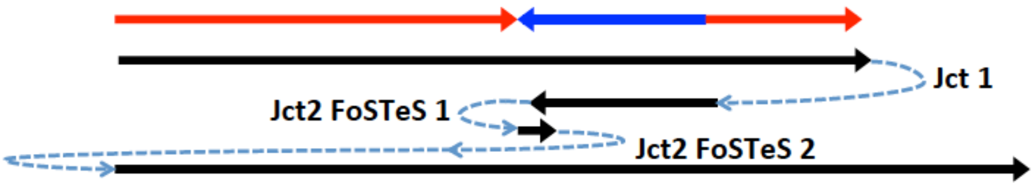

Jct2 for P518

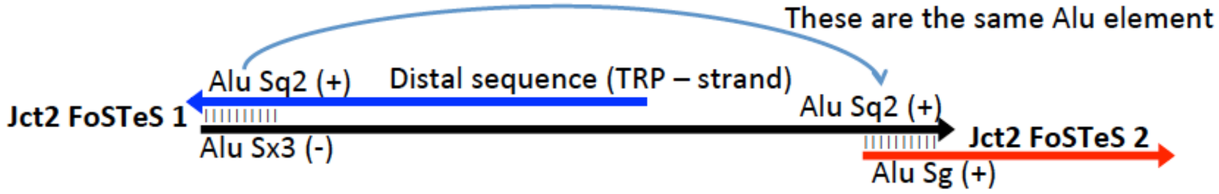

P674

```
Prox.     CATCTCTCTGTCTTCTTCCTCACCCAGAGGGGCTGTGGAGTGAGGATGCATGAGGCCATAATAGCAGGGCAATGGAATCTGACCGTTTCTCAATCCCTGGATGCCCAT
BPT       CAACTTTATCTTTCTGTCCCTACTTCCTGGCTCCTAGCTGCGTTTAGCTACTAGGCCATAATAGCAGGGCAATGGAATCTGACCGTTTCTCAATCCCTGGATGCCCAT
Dis. (-)  CAACTTTATCTTTCTGTCCCTACTTCCTGGCTCCTAGCTGCGTTTAGCTACTAAAGGTTGTCAGCTAACTATTGGAAAATGGACTTTTGGCAGTCTGCCGCATTACTT
```

P820

```
Prox.     TTCTCATAACACTCCTGTGAGGTAAATAGCAATATGCTAATTTTATTGATGAGCAAGGTAAGGTTCACTAAGTGTAACTCACTACCATACCATCATACAATAGAAA
BPT       ACACACCAGGGCCTGTCAGGAGGTGGGGGTAAGGGAGGGATAGCATTAGGAGAAAGGTAAGGTTCACTAAGTGTAACTCACTACCATACCATCATACAATAGAAA
Dis. (-)  ACACACCAGGGCCTGTCAGGAGGTGGGGGTAAGGGAGGGATAGCATTAGGAGAAATACCTAATATAGGTAACGGGTTGATGGGTGTGGCAAACCACCATGGAACA
```

P842

```
Prox.     ATTCCCCTTTCTTTGTTTCTTCCCCAGCATTTTTCTTTTTTTTCTTTCTTTCTTTCTTTTTTTTTTTTTGAGACAGAGTTTCGCTGTCTCCAGGCCGGAGGGCAGTGG
BPT       AGAAAAGGCTTTTCAGCAAATGATATTGGGGCAATTGGATATTCATAAAGAAATTCTTTTTTTTTTTTTGAGACAGAGTTTCGCTGTCTCCAGGCCGGAGGGCAGTGG
Dis. (-)  AGAAAAGGCTTTTCAGCAAATGATATTGGGGCAATTGGATATTCATAAAGAAAACATTGGACCTCTATTCTCATATTCTACATGAAAATCAATTCCAAGTATATTAAA
```

P1150

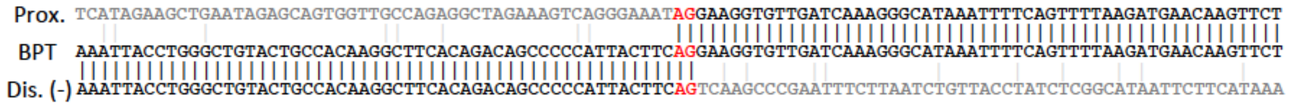

P1150 Deletion

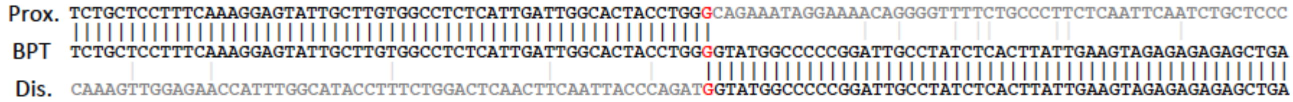

P1389

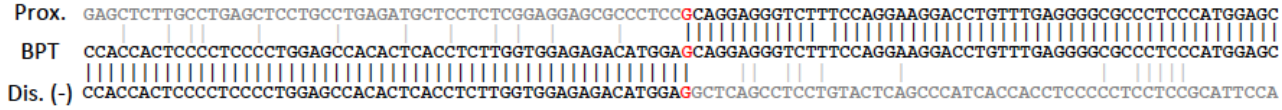

P1407

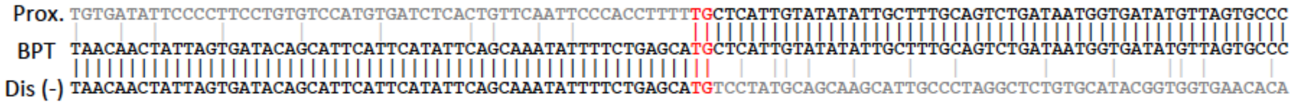

BAB2389

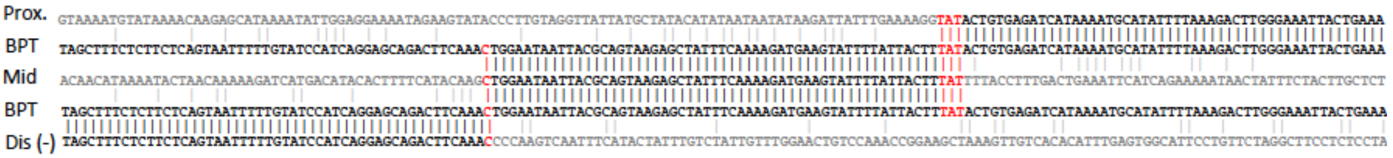

BAB1290

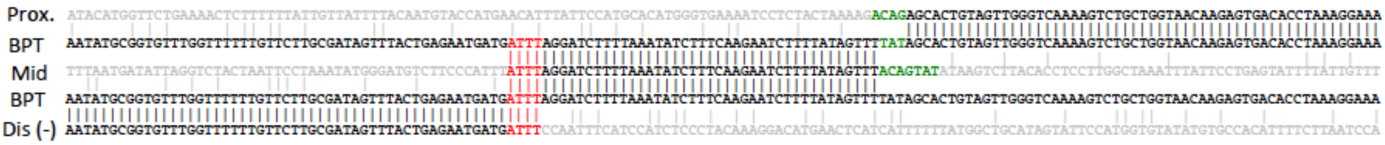

BAB3698- AluSx/AluSx

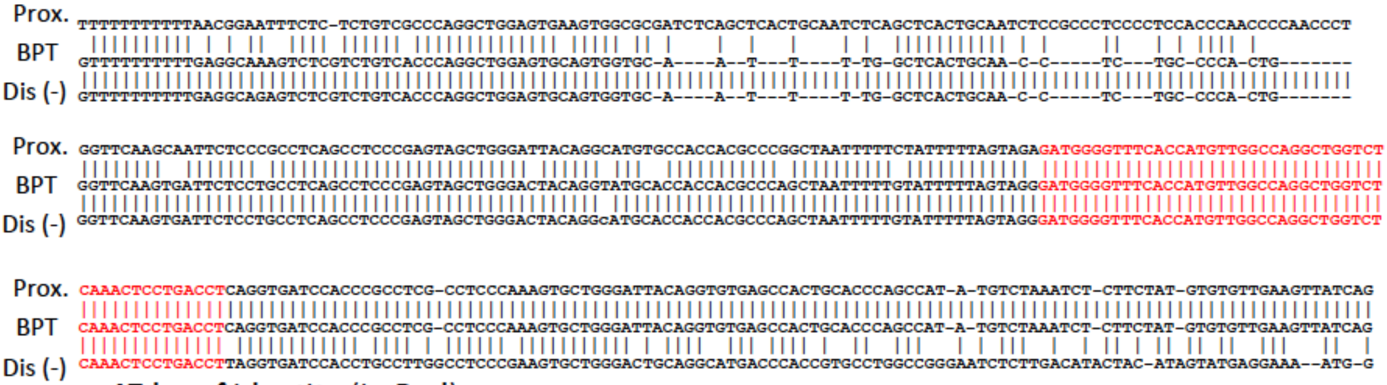

47 bp of identity (in Red)

P113 (Quadruplication)

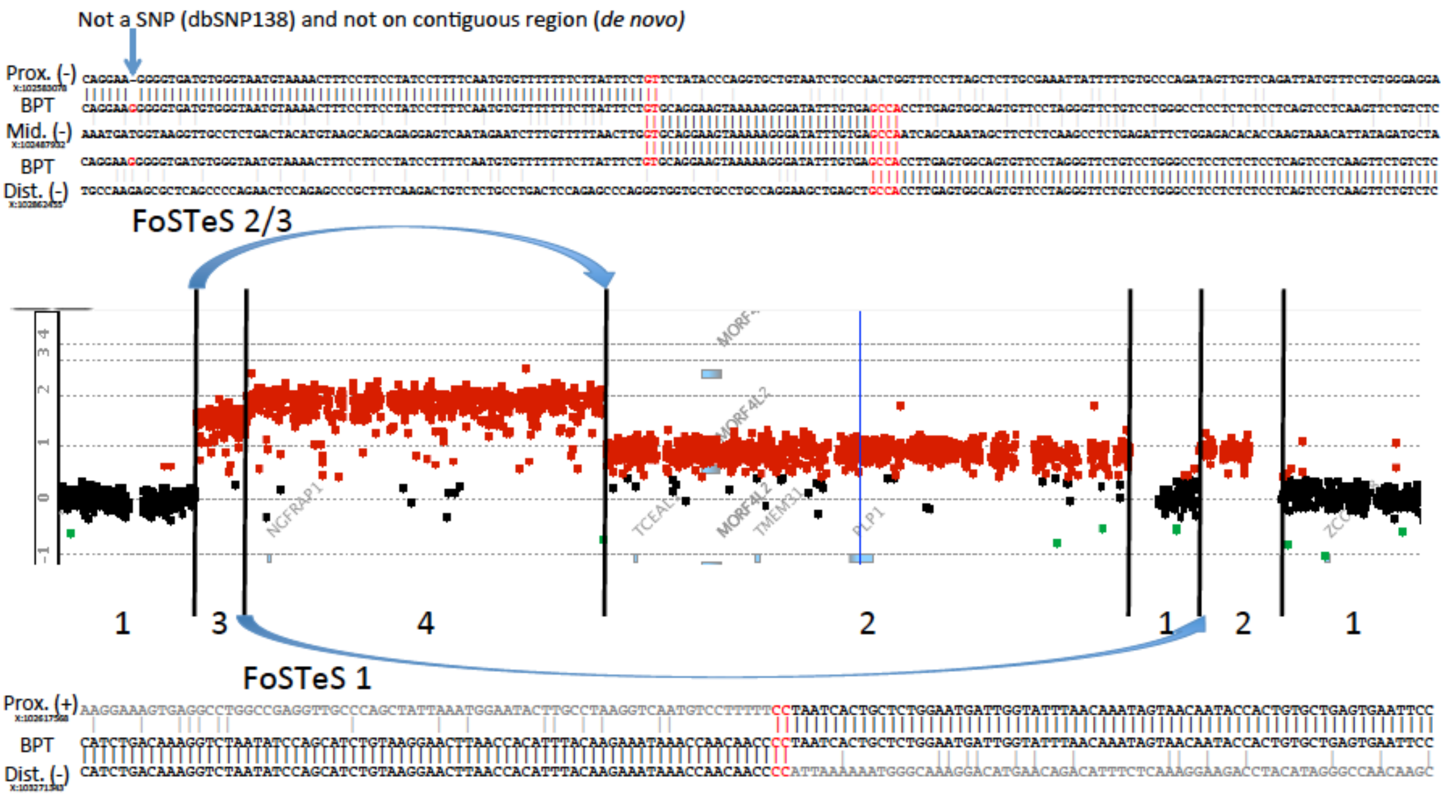

Figure S6- Jct2 and Other Breakpoint Junctions from Patients in the Study
