# Supplementary material for: Complex Genomic Rearrangements at the PLP1 Locus Include Triplication and Quadruplication
Source: PLoS Genet. 2015 Mar 6;11(3):e1005050. doi: 10.1371/journal.pgen.1005050 (PMC4352052; doi:10.1371/journal.pgen.1005050)
Supplement: S8 Fig — To clone the breakpoints from individuals (BAB1612/P374, BAB1290, and BAB2389) with DUP-TRP/INV-DUP rearrangements, the ~20 Kb LCR regions were PCR amplified in two overlapping fragments (relevant inversion allele shown). Black PCR primers anneal to either LCR A1a (blue segments, shortened to “A”) or LCR A1b (purple segments, shortened to “B”), whereas colored primers are unique to the flanking sequences of the LCRs. Red vertical line indicates location of Jct1. Multiple resultant PCR fragments for each region were then cloned and sequenced. Analysis showed varying degrees of homology between the two LCRs in different individuals, and succeeded in cloning the breakpoint from BAB1612/P374. (PDF) [file pgen.1005050.s008.pdf]

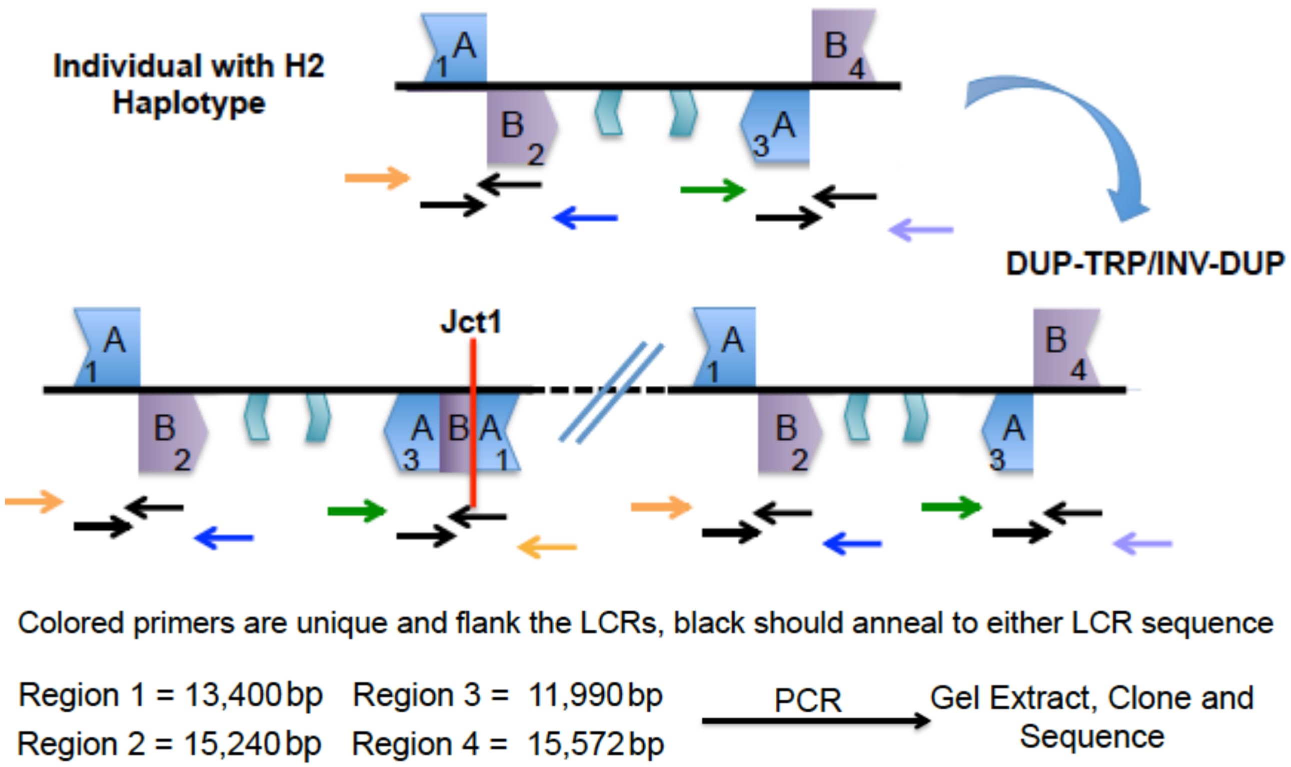

Figure S8- Jct1 Cloning and Sequencing
