# Supplementary material for: Complex Genomic Rearrangements at the PLP1 Locus Include Triplication and Quadruplication
Source: PLoS Genet. 2015 Mar 6;11(3):e1005050. doi: 10.1371/journal.pgen.1005050 (PMC4352052; doi:10.1371/journal.pgen.1005050)
Supplement: S1 Table — Southern blot genotyping of the inversion in 17 HapMap individuals was quantitated using GelAnalyzer 2010 software. Briefly, lanes and bands were designated according to the histograms, and normalization was determined automatically by the valley-to-valley normalization function, Resultant band intensities (Raw Volume) were used to generate ratios of the bands if more than one was present. This ratio is represented in the final column of the table, and heterozygous HapMap females have an expected ratio of 1. Asterisks indicate DNAs that were on both blots. (PDF) [file pgen.1005050.s012.pdf]

**Blot from Figure 1**				
**Individual**	**Lane #**	**Band#**	**Raw Volume**	**Ratio**
NA15510*	1	1	66	1.1
	1	2	61	
NA18555	2	1	99	0.92
	2	2	108	
NA18942	3	1	244	NA
NA18947	4	1	180	NA
NA18956	5	1	396	NA
NA19129	6	1	141	0.85
	6	2	165	
NA19240*	7	1	41	0.98
	7	2	42	
NA18707	8	1	266	NA
NA18873	9	1	87	1.02
	9	2	85	
**Blot from Figure S3**				
Individual	Lane #	Band#	Raw Volume	Ratio
NA15510*	1	1	30	1.3
	1	2	23	
NA10851	2	1	221	NA
NA11993	3	1	179	NA
NA12156	4	1	53	0.85
	4	2	62	
NA12878	5	1	138	NA
NA18507	6	1	118	NA
NA18517	7	1	132	NA
NA19240*	8	1	83	0.88
	8	2	94	
NA18552	9	1	269	NA
NA18502	10	1	161	NA

Table S1- Semi-Quantitative Analysis of HapMap Southern Blots
