# Supplementary material for: Complex Genomic Rearrangements at the PLP1 Locus Include Triplication and Quadruplication
Source: PLoS Genet. 2015 Mar 6;11(3):e1005050. doi: 10.1371/journal.pgen.1005050 (PMC4352052; doi:10.1371/journal.pgen.1005050)
Supplement: S2 Table — Southern blot genotyping of the inversion in 4 HapMap populations, as well as one individual of unknown population of origin. (PDF) [file pgen.1005050.s013.pdf]

Population of Origin	H1 (Reference)	H2 (Inversion)
Han Chinese	2	3
Japanese	4	2
Yoruban	8	3
CEPH	3	4
Unknown	1	1
Total	18	13

Table S2- Population Distribution of H1 and H2
