# Supplementary material for: Complex Genomic Rearrangements at the PLP1 Locus Include Triplication and Quadruplication
Source: PLoS Genet. 2015 Mar 6;11(3):e1005050. doi: 10.1371/journal.pgen.1005050 (PMC4352052; doi:10.1371/journal.pgen.1005050)
Supplement: S3 Table — (PDF) [file pgen.1005050.s014.pdf]

Patient	CA-PLP2/3 CA repeat	c.5-411T>C	c.5-111T>C	c.609T>C	839M11-F24/R24 A/C	839M11-F25/R25 A/C	839M11-F26/R26 A/G	U240C2-F19/R19 T/A	U240C2-F20/R20 G/A	U240C2-F21/R21 T/G	U240C2-F18/R18 CA repeat
P0250	182bp	C	C	T	C	C	G	T	G	T	214/18
P0255/P0298	192bp	T	C	T	C	C	G	T	G	T	212/17
P0374*	ND	ND	ND	ND	C	C	G	T	G	T	219/21;223/23
P0500	180bp	C	C	C	C	C	G	T	G	T	216/19
P0518	182bp	T	T	T	C	C	G	T	G	T	216/19
P0558	184bp	C	C	C	A	A	A	A	A	G	216/19
P0642	180bp	C	C	C	C	C	G	T	G	T	212/17
P0674	182bp	T	T	T	A	A	A	A	A	G	216/19
P0820	182bp	T	T	T	C	C	G	T	G	T	216/19
P0842	184bp	T	T	T	A	A	A	A	A	G	216/19
P1150	182bp	T	T	T	C	C	G	T	G	T	216/19
P1389	192bp	T	T	T	A	A	A	A	A	G	216/19
P1407	180bp	C	C	C	A	A	A	A	A	G	216/19
											bp/repeats
position on NT_011651.17	26331677	26336408	26336708	26339190	26548289	26557893	26570534	26577150	26584297	26591851	26590136

* P0374 is not duplicated at PLP1, so no analysis was done in the PLP1 region.

Table S3- Results of SNP and STR Genotyping
