# Supplementary material for: Complex Genomic Rearrangements at the PLP1 Locus Include Triplication and Quadruplication
Source: PLoS Genet. 2015 Mar 6;11(3):e1005050. doi: 10.1371/journal.pgen.1005050 (PMC4352052; doi:10.1371/journal.pgen.1005050)
Supplement: S4 Table — Southern blot genotyping of DUP-TRP/INV-DUP patients was quantitated using GelAnalyzer 2010 software, as in S1 Table. This ratio is represented in the final column of the table. Ratios of ~2 in the patients indicate H2 rearrangements, while less than one indicate H1 (expected ratio of ~0.5). Carrier females from BAB3698 family and females without rearrangements have expected ratios of ~1. (PDF) [file pgen.1005050.s015.pdf]

Blot from Figure 3B				
Individual	Lane #	Band#	Raw Volume	Ratio
NA15510	1	1	43	1.05
	1	2	41	
NA10851	2	1	75	NA
BAB1290	3	1	91	2.17
H2	3	2	42	
BAB1612	4	1	50	2.38
H2	4	2	21	
**Blot from Figure 3C**				
**Individual**	**Lane #**	**Band#**	**Raw Volume**	**Ratio**
NA15510	1	1	516	1.24
	1	2	417	
BAB2389	2	1	865	2.21
H2	2	2	391	
BAB4179	3	1	1011	NA
BAB3700	4	1	584	1.21
	4	2	483	
BAB3698	5	1	1145	2.23
H2	5	2	512	
BAB3699	6	1	549	1.06
	6	2	516	
**Blot from Figure 3D**				
**Individual**	**Lane #**	**Band#**	**Raw Volume**	**Ratio**
NA15510	1	1	74	0.81
	1	2	91	
P250	2	1	54	0.13
H1	2	2	426	
P298	3	1	117	0.45
H1	3	2	261	
P500	4	1	181	1.92
H2	4	2	94	
P518	5	1	122	1.87
H2	5	2	65	
P558	6	1	41	0.32
H1	6	2	129	
P642	7	1	82	1.74
H2	7	2	47	

Table S4- Semi-Quantitative Analysis of Patient Southern Blots
