# Supplementary material for: Complex Genomic Rearrangements at the PLP1 Locus Include Triplication and Quadruplication
Source: PLoS Genet. 2015 Mar 6;11(3):e1005050. doi: 10.1371/journal.pgen.1005050 (PMC4352052; doi:10.1371/journal.pgen.1005050)
Supplement: S5 Table — The table depicts digital PCR results from Jct/FoSTeS 1 and Jct/FoSTeS 2/3 in patient P113, the affected uncle of the proband P117, and the carrier mother of P113, P154. In each experiment, FoSTeS 1 has a copy number of 1, and FoSTeS 2/3 has a copy number of 2. Pooled normal control individuals (6NLs) have neither junction. *Copies/genome is calculated for each junction as (copies/microliter jct)/([copies/microliter RNaseP]/2). Note that RNase P is autosomal and so has two copies per male or female genome (one on each allele). The junctions are located on the X chromosome; there is only one X allele in males, and it is affected in the patients. Female carriers have junctions on one X allele but not the other. (PDF) [file pgen.1005050.s016.pdf]

Experiment 1									
Target	Sample	**Copies/ genome***	Copies/ microliter	CI Copies/ microliter	Copies /Rxn	CI Copies/ Rxn	# of Neg	# qualified by QT	# of Filled
Jct 1	P113_1	1.14	283.74	274.84 -- 292.93	0.245	0.238 -- 0.253	13664	17465	18060
RnaseP	P113_1		499.67	487.22 -- 512.44	0.432	0.421 -- 0.443	11336	17465	18060
Jct 1	P117_1	1.00	286.61	277.86 -- 295.63	0.248	0.240 -- 0.256	14281	18299	18505
RnaseP	P117_1		570.96	557.73 -- 584.51	0.494	0.482 -- 0.506	11167	18299	18505
Jct 1	P154_1	1.11	321.31	311.84 -- 331.07	0.278	0.270 -- 0.286	13477	17795	18479
RnaseP	P154_1		580.31	566.75 -- 594.19	0.502	0.409 -- 0.514	10772	17795	18479
Jct 2/3	P113_2/3	2.04	582.18	568.62 -- 596.07	0.504	0.492 -- 0.516	10786	17847	18097
RnaseP	P113_2/3		569.50	556.12 -- 583.19	0.493	0.481 -- 0.504	10905	17847	18097
Jct 2/3	P117_2/3	2.00	579.08	565.69 -- 592.79	0.501	0.489 -- 0.513	11021	18187	18548
RnaseP	P117_2/3		578.45	565.07 -- 592.15	0.500	0.489 -- 0.512	11027	18187	18548
Jct 2/3	P154_2/3	2.03	578.93	565.26 -- 592.92	0.501	0.489 -- 0.513	10577	17452	18621
RnaseP	P154_2/3		571.63	558.07 -- 585.51	0.494	0.483 -- 0.506	10644	17452	18621
**Experiment 2**									
Target	Sample	**Copies/ genome***	Copies/ microliter	CI Copies/ microliter	Copies /Rxn	CI Copies/ Rxn	# of Neg	# qualified by QT	# of Filled
jct1	P113_1	1.08	487.66	475.45 -- 500.18	0.422	0.411 -- 0.433	11560	17626	17677
RNAseP	P113_1		905.01	886.60 -- 923.80	0.783	0.767 -- 0.799	8057	17626	17677
jct1	6NLs_1	0.05	32.475	29.685 -- 35.528	0.028	0.026 -- 0.031	16708	17184	18635
RNAseP	6NLs_1		1209.9	1186.6 -- 1233.6	1.047	1.026 -- 1.067	6034	17184	18635
jct2/3	P113_2/3	2.11	909.59	891.46 -- 928.09	0.787	0.771 -- 0.803	8337	18311	18334
RNAseP	P113_2/3		860.72	843.29 -- 878.51	0.745	0.729 -- 0.760	8697	18311	18334
jct2/3	6NLs_2/3	0.00	2.277	1.601 -- 3.238	0.002	0.001 -- 0.003	15723	15754	18501
RNAseP	6NLs_2/3		1210.1	1185.8 -- 1234.9	1.047	1.026 -- 1.068	5531	15754	18501

Table S5- dPCR Results for FoSTeS Jct 1 and 2/3 from Quadruplication
