# Supplementary material for: Complex Genomic Rearrangements at the PLP1 Locus Include Triplication and Quadruplication
Source: PLoS Genet. 2015 Mar 6;11(3):e1005050. doi: 10.1371/journal.pgen.1005050 (PMC4352052; doi:10.1371/journal.pgen.1005050)
Supplement: S6 Table — SNP primers amplify de novo nucleotide changes, and Inv. PCR primers were used in inverse PCR of sample P1150. Additional primers are available upon request. (PDF) [file pgen.1005050.s017.pdf]

Jct Primers

Patient	Jct2 Duplication Reverse	Jct2 Triplication Reverse
P250	TGGGGACATTGACCAAATATATACTGTAAT	TAGGATGACTGCTTTTTGCTCAA
P255	AACATCGAAATATTTCCACTTACTTGGTT	AAGACTCCAAAGGCAAGGCA
P500	AGTAGCCAATATGGAAAGAAGGTCT	AAGTGCCAGTAGCCTCTGAGTT
P518	AACGTTGCCATTGCTGTATCAACTAA	TGCTCACTACCACTTCCCTCTCAA
P558	ATGTTGGCCAGGCTGGTCT	AAGGGATCAATTCAACAAGAGGAGCTA
P642	AAGCTGAGAAGCCACTGTGATGT	ATGGTTGGTTGGTCAGACATGAT
P674	AACACCAGTCTATGTCTTATATTGTTATGGCTA	ACTGTGCATTCCCTCCTTAGCGT
P820	TGTGTCATTCACAGTGCCAGTGTT	AGGGACATGGATGATGCCTGAA
P842	CTGGGGAAGGCCTGGCTT	TCTTGAGGAATAAGAATGAGGTGGGAA
P1150	TACCAGAGAGAATATCTGGAAATGTAACACCAA	TGCACCTTATCAACCAAATTATCTTGCCTA
P1389	ATGCAAATGTGTAGGGCTCATGA	AGGCCAGGAAGCAGATGGCTA
P1407	TTTGAAAGATGGTCATAGTAAAGGCAA	ACAACTAGTGTTAGTCATGTTGCTTCCAT
BAB 1290	GAAGATGAGGGAAACCAGGAA	ATCATTGAGACTACTGAAAATGGATTTACATA
BAB 2389	ATGAGTGATCATTATCTATATGAATTGTTATCCTT	ATCTCCGACTTAGGATTTCAGCATA
BAB 3698	ATCCAGGTTCCACTATTTACAAGTTATATGA	ATACGACGTGTATCAGAGTCACCA

Patient/BPT	Forward	Reverse
P113 TRP/QUAD	AGTAGAGCATCATTGTCACAGTTATTAAGGAA	TGCAGTTGCCGGTGAAGTCTT
P113 QUAD/DUP	TGCAGTTGCCGGTGAAGTCTT	AGTCCTTTCCAGTATTACCATAGCTACTCTTAGC
P1150 deletion	TTGGCCCATCTGGACAAGATCTATAA	AAGATGCAGGCTTGCTCCTTAGAGAT
P113 SNP	TTAATTGAGATGTGAGGAGCTAGATGTCA	AGTAGAGCATCATTGTCACAGTTATTAAGGAA
P250 SNP	TTGTAAGAGAGCAACTAGGTTCCAT	TTAGAGATTTCTACAAGAGTAGAGCTGTAGTTA

Jct1 primer name	Sequence
Hdys 23F-6FAM	ATTGAGGGACGCTGGAAGAAGC
Hdys 23R	GGAATAAGCAAATCGCCATCC
V362H12-F19-6FAM	AAGGCTGGAGTTAAATGATTGTC
V362H12-R19	TTATTCTTTGCAGAGATGAATTAGG
V362H12-F24-6FAM	ACATGCAGGTGTGTTATACA
V362H12-R24	ACAAAAAAACCGAATGCCACA

SNP and STR Amplicon Primers-

SNP/indel			Primer sequence			
rs#		position on NT_011651.17	forward	position on NT_011651.17	reverse	position on NT_011651.17
rs72325892*	CA-PLP2/3	26331693	CCCAATGCTTGCACATAAATTG	26331623-26331644	AATCTCAGCCTCCTCTTTGGACAC	26331801-26331778
rs508804	c.5-411T>C	26336408	TAACTGAGGGTGGGGACATTAGCC	26335606-26335629	**CAAGCCTGGGGAAGAAGAAGGG**	26336823-26336802
rs2233696	c.5-111T>C	26336708				
rs1126707	c.609T>C	26339190	ATTCCCCCCACCCTCCGTTATACTG	26338821-26338845	**GGTTGTGAACAGCAGTCAACTAGAG**	26339453-26339429
rs479295	839M11-F24/R24	26548289	GGGGCTTAGGGTATGAAGCTG	26548261-26548281	**AAAGACAGTTGTGGGTCTGGTATG**	26548467-26548444
rs479400	839M11-F25/R25	26557893	TAAGACCCAAACAGACGCTCTAATG	26557740-26557764	**CATAGGCAAGTGAGCACATAAGTTC**	26558078-26558054
rs494006	839M11-F26/R26	26570534	CCATTCAACTTTCATACTTTGGCG	26570435-26570458	**TTCTGATGTCAGGCATCCTGTTTC**	26570698-26570675
rs4826897	U240C2-F19/R19	26577150	**GGAAGATGACTGGGACTGAAACAC**	26577438-26577415	TATGTTCTCTCTCCACAGAAGTGAG	26577007-26577031
rs1024326	U240C2-F20/R20	26584297	CAAAAGAGCACAATTGGAGGTGC	26584392-26584370	**TACTTAAATCGCAAGGATGCCACC**	26584195-26584218
rs3220164*	U240C2-F18/R18	26590136	GTTGATACCCATTGAAGGATGCAC	26590270-26590247	TAGTAATGTTTGCCCCGTTTGATG	26590072-26590095
rs3213517	U240C2-F21/R21	26591851	**TCAGGTAAAGAATCACACTGCCG**	26592184-26592162	TGTGTTCAGCATCTCTCTCATCATG	26591776-26591800

* product size(s) analyzed by capillary electrophoresis

primers in **bold** used for sequencing

Additional Jct2 Primers

Patient		Primer	Accession	Position on Clone	Sequence	Chr.X Start (hg19)	Chr.X End (hg19)
**P0250**	PCR/seq	857G6-F14	Z73965	1938-1961	TCAAAAACTCAAGGCTCCTGACTC	102866307	102866284
	PCR	370B6-R	AL390022	7083-7061	CTTCCTCTGAGATGACCTCCAAC	103159630	103159608
**P0255**	PCR	1055C14-R10	AL049610	72119-72096	TGCCAGGAATTATCCAGGTTCCAC	102945132	102945109
	PCR	467E10-F3	Z74620	5005-5028	TGGTAAAGTTGAGGACTTAATTGC	103322760	103322783
	seq	1055C14-R18	AL049610	70832-70813	AAAATCCCTCCTGTGTGGTG	102943845	102943826
**P0374**	PCR	V362H12-R21	Z70227	13219-13195	CTTTGCTCGTCTTTTCTCACTAAGG	103201518	103201494
	PCR	U116E7-R11	Z70273	19181-19162	CCCTGCTCCATCCATTTTCC	103180905	103180886
	seq	U116E7-R13	Z70273	18393-18374	GAGGGGGTTGACATGGGGGC	103180117	103180098
**P0500**	PCR	540A13-R10	AL139228	7394-7370	AGAAGGAAGGGATAATGACAAAAGC	103074666	103074642
	PCR	421I20-R15	AL117327	43550-43530	TGCCCCACCAAGGGTTAGGGA	102726879	102726859
	seq	540A13-R20	AL139228	6927-6908	ACCTGCCAAGCTGTGCCACA	103074199	103074180
**P0518**	PCR	U35G3-R16	Z93848	31742-31721	CCTGCTCACTACCACTTCCCTC	103004927	103004906
	PCR	198P4-R3	AL008708	34046-34023	TATTATGAGGCTTGTCCCTGTGTC	102327890	102327867
	seq	U35G3-R17	Z93848	31275-31256	GATTACAGGCATCCACCACC	103004460	103004441
	seq	198P4-R12	AL008708	31018-30999	GATGGAGTCTTGCTCTGTCG	102324862	102324843
**P0558**	PCR	246D9-R3	AL021308	3238-3215	TATGTATTACGCTATGACACAGGG	102807374	102807351
	PCR	540A13-R23	AL139229	15726-15707	GGCACAGGCCAGTAGGTGCG	103152323	103152304
	seq	540A13-R22	AL139229	13983-13964	GGAACAACCGGTACCAGCCG	103150580	103150561
**P0642**	PCR/seq	349O20-R12	AL606763	11568-11547	ATCAGTGGAAGCTCGTTTTTGC	102627776	102627755
	PCR/seq	349O20-R7	AL606763	32614-32591	GGTGGGCAAAGAATGTCTGTCTTC	102648822	102648799
**P0674**	PCR	43H13-R4	AL035444	17341-17318	TATACTGATGACTGTGGATGGGGC	102794098	102794075
	PCR	1055C14-R23	AL049610	91405-91386	CCAACTTTCC***C***CCTCAGAGC	102964418	102964399
	seq	1055C14-R27	AL049610	90684-90663	GCAGAGGTCCCACGATAAAGAC	102963697	102963676
**P0820**	PCR/seq	U250H12-F9	Z69733	6062-6085	ACAGTGCCAGTGTTTGCTTCCTTC	102770796	102770773
	PCR	857G6-R	Z73965	16168-16145	AAGCTCTGAGATGTAGAGAGCAGG	102852077	102852100
**P0842**	PCR	349O20-R6	AL606763	27224-27201	CTGGCATTGAAGAAAATAGAACAG	102643432	102643409
	PCR	V362H12-R8	Z70227	9921-9898	GGTGTCAGATTATGGAGGACCTTG	103198220	103198197
	seq	V362H12-R26	Z70227	9368-9347	CTCAGAGTTGTCCCATTTGTAC	103197667	103197646
**P1150**	Inv. PCR	764D10-R3	AL034409	11288-11266	TCACCATCAGTCATCCAGCAAAG	103024169	103024147
	Inv. PCR	246D9-R2	AL021308	310-290	CCCTCAAGTTGACCTCAGTCC	102804446	102804426
	seq	43H13-R11	AL035444	27228-27205	ATTGCTTGAACCTGGGAGGCAGAG	102803974	102803951
	PCR	U35G3-R12	Z93848	14433-14409	CAAGTGGCTGATGTGATGAGTAACG	102987618	102987594
	PCR/seq	1055C14-F25	AL049610	86664-86687	GGAAGGGTGAGGAAAATGAAAGTC	102959677	102959700
**P1389**	PCR/seq	U116E7-R11	Z70273	19181-19162	CCCTGCTCCATCCATTTTCC	103180905	103180886
	PCR	1055C14-R35	AL049610	15418-15394	CATCTGCTTGCTCAAAGACTATCCC	102888431	102888407
**P1407**	PCR/seq	540A13-R17	AL139228	21933-21909	CGTGCTGTCCATTTTTGCTGATCCC	103089205	103089181
	PCR	U250H12-F2	Z69733	29157-29180	ACTACCTTGATGAGTGTCAGTGTG	102747701	102747678

Probe/Primer Sequences for dPCR assays

jct1	
Probe	/56-FAM/CCC TAA TCA /ZEN/CTG CTC TGG AAT GAT TGG T/3IABkFQ/
Reverse primer	CTC AGC ACA GTG GTA TTG TTA CT
Forward primer	GGT CTA ATA TCC AGC ATC TGT AAG G
jct2/3	
Probe	/56-FAM/TAT TTG TGA /ZEN/GCC ACC TTG AGT GGC A/3IABkFQ/
Reverse primer	GGA CAG AAC CCT AGG AAC A
Forward primer	GGG TGA TGT GGG TAA TGT AAA

Table S6- Primers and Probes Used in the Manuscript
